# Clonal *LIMD1* loss drives PD-L1 immune evasion via ARIH1-dependent ubiquitination in lung cancer

**DOI:** 10.26508/lsa.202603812

**Published:** 2026-08-07

**Authors:** Kunal M Shah, Paul T Kennedy, James RM Black, Piotr Pawlik, Kevin Litchfield, Krupa Thakkar, Maria F Contreras-Gerenas, Kirsten Brooksbank, Oliver Yuan, Paul Grevitt, Sarah Charrot, Jeff Davies, Lekh N Dahal, Dimitris Lagos, Nicholas McGranahan, Tyson V Sharp

**Affiliations:** 1 The Centre for Cancer Cell and Molecular Biology, Barts Cancer Institute, Queen Mary University of London, John Vane Science Centre , London, UK; 2 Department of Pharmacology and Therapeutics, University of Liverpool , Liverpool, UK; 3 North West Cancer Research Institute, North Wales Medical School, Bangor University, Bangor, UK; 4 UCL Cancer Institute , London, UK; 5 Hull York Medical School, University of York , Heslington, York, UK; 6 The Centre for Haemato-Oncology, Barts Cancer Institute, Queen Mary University of London, John Vane Science Centre , London, UK

## Abstract

LIMD1, a tumour suppressor located at chromosome 3p21.3, is frequently lost in non–small-cell lung cancer, yet its role in tumour–immune interactions remains unclear. Here, we show LIMD1 loss increases PD-L1 protein abundance across multiple lung cancer models and primary airway epithelial cells. Mechanistically, LIMD1 restrains PD-L1 through post-transcriptional and post-translational mechanisms. LIMD1 loss can relieve microRNA-mediated repression of the *CD274* 3′UTR, and LIMD1 loss can also disrupt ARIH1-PD-L1 association, reduce PD-L1 polyubiquitination, and stabilise PD-L1 protein without a commensurate increase in *CD274* transcript levels in isogenic models. Functionally, LIMD1-deficient tumour cells suppress CD8^+^ T-cell activation in vitro and show enhanced sensitivity to PD-1/PD-L1 blockade in tumour–PBMC co-culture assays. Analysis of TRACERx non–small-cell lung cancer samples revealed clonal LIMD1 loss of heterozygosity in ∼40% of lung adenocarcinomas, where it is associated with increased tumour PD-L1 expression. Across independent patient cohorts receiving immune checkpoint blockade, low LIMD1 expression was enriched among responders. We identify LIMD1 as a tumour-intrinsic regulator of PD-L1 turnover and suggest that tumour suppressor loss can shape immune checkpoint biology and influence immunotherapy response.

## Abstract (precis)

Lung cancer tumour suppressor LIMD1 regulates the expression of a key immune checkpoint protein PD-L1. This occurs via loss of miRNA-mediated regulation of PD-L1 or ARIH1-dependent PD-L1 ubiquitination. Low LIMD1 expression was enriched among responders to immune checkpoint blockade.

## Introduction

Lung cancer is responsible for 21% of total cancer deaths, with ∼35,000 deaths annually in the UK. This high mortality rate is attributed to late diagnosis and frequent therapeutic resistance. Although immune checkpoint blockade (ICB) therapies have transformed cancer treatment, response rates remain variable, typically around 20–40% (Hirsch et al, 2017). Enhancing these response rates necessitates a comprehensive understanding of the molecular mechanisms governing immune checkpoint protein expression.

The programmed death-1/programmed death ligand-1 (PD-1/PD-L1(*CD274*)) axis is an immunosuppressive pathway that regulates cytotoxic T-cell responses, preventing tissue damage and maintaining immune tolerance. PD-L1 signalling counteracts T-cell receptor stimulation, reducing T-cell proliferation and cytotoxicity. Cancer cells exploit this pathway by aberrantly expressing PD-L1, disrupting immunosurveillance, and facilitating immune evasion.

ICB therapies targeting the PD-1/PD-L1 axis have become widespread and highly effective because of rescue of the antitumour effector response. However, a significant proportion of patients do not respond to these treatments, whereas some suffer adverse effects. Despite challenges because of the dynamic nature of PD-L1 expression within tumours and inconsistencies in diagnostic pathology methods, it has become evident that high PD-L1 expression is associated with better response to ICB in lung cancer (Buttner et al, 2017; Hirsch et al, 2017; Gadgeel et al, 2022; John et al, 2024). Thus, studying the cellular and genetic processes that regulate PD-L1 expression is an active research area aimed at improving response rates and patient outcomes.

Tumour PD-L1 expression involves critical transcriptional processes, including JAK-STAT-IRF1 signalling activated by interferon stimulation, Ras-MEK-ERK signalling triggered by receptor tyrosine kinase activation (e.g., EGFR signalling), and NF-κB activation (Lee et al, 2006; Jiang et al, 2013; Gowrishankar et al, 2015). Epigenetic modifications at the *CD274* locus also influence gene transcription (Woods et al, 2015; Buttner et al, 2017; Deng et al, 2019; Frankell et al, 2023). Post-transcriptionally, RNA-binding proteins and microRNAs can negatively affect PD-L1 mRNA stability and translation (Chen et al, 2014; Coelho et al, 2017; Yee et al, 2017), whereas PD-L1 mRNA translation can be promoted through the integrated stress response (Suresh et al, 2020). Post-translational modifications, such as glycosylation, phosphorylation, ubiquitination, acetylation, and palmitoylation, are known to regulate PD-L1 protein stability and interactions (Koyama et al, 2016b; Jagannathan et al, 2016; Deng et al, 2019; Yao et al, 2019; Gao et al, 2020; Zhao et al, 2022).

Understanding how mutations in tumour suppressors and oncogenes trigger PD-L1 expression and facilitate immune evasion in distinct cancer types is crucial to improving response rates and patient outcomes. This knowledge can refine patient stratification for PD-1/PD-L1 ICB therapies, improving their predictability and efficacy (Koyama et al, 2016b; Peng et al, 2016).

Recognised as a tumour suppressor, LIMD1 encodes a 72-kD protein with three LIM domains at its C terminus, part of the zyxin family (Siddiqui et al, 2021). LIMD1 regulates hypoxia-inducible factor-1α (HIF-1α) and the Rb-E2F1 complex, influencing cell-cycle progression and post-transcriptional gene regulation via microRNA-mediated gene silencing (Sharp et al, 2004; James et al, 2010; Foxler et al, 2012, 2018; Bridge et al, 2017; Crozier et al, 2026). LIMD1 also interacts with the Hippo signalling pathway, emphasising its role in diverse cellular processes (Das Thakur et al, 2010; Jagannathan et al, 2016). Loss of LIMD1 is common in non–small-cell lung cancer (NSCLC) (Foxler et al, 2018), but its implications for tumour–immune interaction remain unknown. Here, we show that LIMD1 loss is clonal in a significant proportion of lung cancers. We investigate the impact of LIMD1 loss on tumour immunosurveillance, focusing on PD-L1 regulation. Via a novel interaction with ARIH1, we find that LIMD1 is a critical regulator of PD-L1 protein turnover and restricts PD-L1 expression to maintain robust cancer immune surveillance. LIMD1 also regulates PD-L1 expression via the 3′-untranslated region (UTR) of *CD274* mRNA in keeping with LIMD1’s established role in the microRNA pathway (James et al, 2010; Bridge et al, 2017).

We report increased PD-L1 checkpoint activity, reduced T-cell activation, and enhanced sensitivity of LIMD1-deficient tumour cells to PD-1/PD-L1 blockade in PBMC co-culture assays. Notably, tumours with low LIMD1 expression exhibit enhanced sensitivity to immune checkpoint inhibitors (ICIs), underscoring the potential of LIMD1 as a biomarker for patient stratification in immunotherapy. Thus, LIMD1 is a crucial and previously unidentified regulator of PD-L1, highlighting therapeutic implications for lung cancer patients with LIMD1-deficient tumours.

## Results

### LIMD1 loss increases PD-L1 through distinct post-transcriptional and post-translational mechanisms

**Figure 1.**
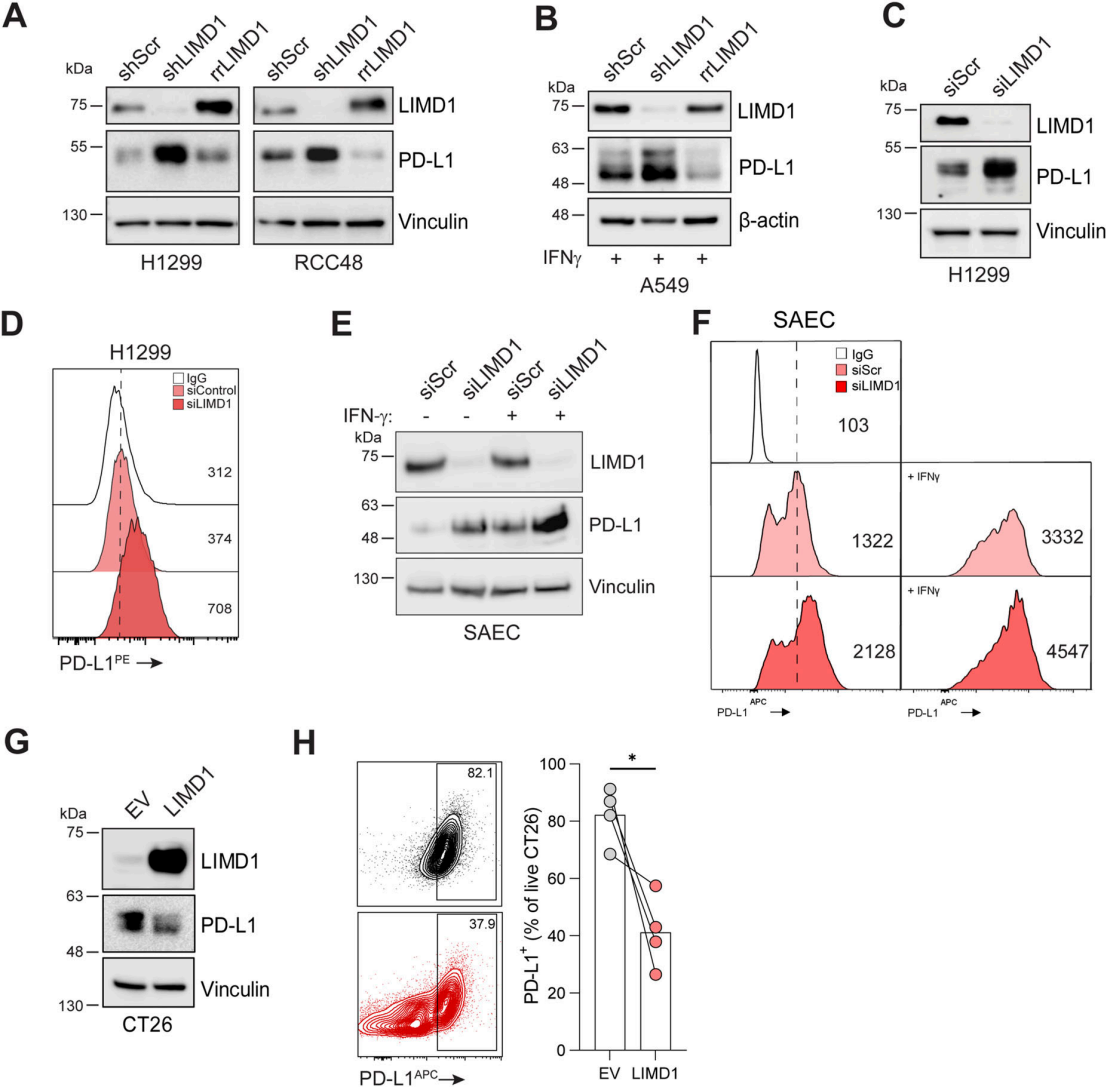
LIMD1 regulates PD-L1 expression in multiple primary and transformed lung cell types. **(A, B)** Immunoblots of PD-L1 protein in H1299, RCC48, and A549 cells transduced with shRNA for LIMD1 and RNAi-resistant LIMD1 lentiviral vectors. A549 cells were stimulated with IFNγ (2 ng/ml) overnight. **(C, D)** Immunoblot and flow cytometric analysis showing PD-L1 expression in H1299 cells after LIMD1 silencing with siRNA (20 nM). **(E)** Immunoblot of PD-L1 and LIMD1 levels in SAEC whole-cell lysates after LIMD1 knockdown with siRNA and treatment with IFNγ (0.5 ng/ml) for 24 h. **(F)** Flow cytometric analysis of surface PD-L1 in SAEC transfected with siScr or siLIMD1. Cells were treated with IFNγ (0.5 ng/ml) or BSA control for 24 h. Values indicate median fluorescence intensity. **(G)** Immunoblot showing stable LIMD1 overexpression and PD-L1 reduction in CT26 colorectal carcinoma cells, with vinculin probed as a loading control. **(H)** Flow cytometric analysis of surface PD-L1 in CT26. Data represent three independent replicates (*P < 0.05, paired *t* test). Source data are available for this figure.

**Figure. S1.**
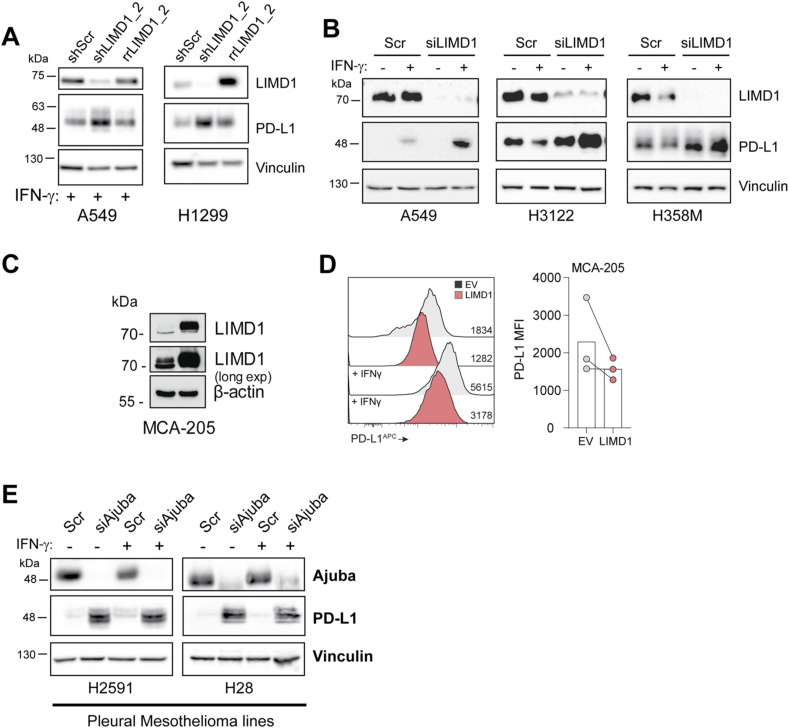
PD-L1 is upregulated in NSCLC lines by LIMD1 depletion and in mesothelioma lines by Ajuba depletion. **(A)** Immunoblots for A549 and H1299 lentiviral lines expressing a second shLIMD1 sequence (shLIMD1_2 and rrLIMD1_2). A549 cells were treated overnight with 2 ng/ml IFNγ. Vinculin was probed as a loading control. **(B)** Immunoblots of PD-L1 in lung adenocarcinoma cells transfected with LIMD1-targeting or scrambled control siRNA (20nM) 48 h before stimulating with IFNγ (2 ng/ml) overnight. Vinculin was probed as a loading control. **(C)** Stable LIMD1 overexpression in MCA-205 fibrosarcoma cells. **(D)** Flow cytometric analysis of surface PD-L1 in MCA-205. **(E)** Ajuba knockdown in mesothelioma cell lines up-regulates PD-L1 protein. H2591 and H28 cell lines were transfected with Scr siRNA or Ajuba siRNA and treated with or without IFNγ. Whole-cell lysates were immunoblotted for PD-L1, Ajuba, and vinculin as a loading control.

We initially examined how PD-L1 expression is affected by LIMD1 depletion in several cancer cell lines. LIMD1 depletion with short hairpin RNA (shRNA) up-regulated PD-L1 protein in a range of cancer cell types, including H1299, A549, and RCC48 carcinoma cells (Fig 1A and B). Re-expression of an RNAi-resistant LIMD1 cassette rescued control of PD-L1 expression and reduced it to levels comparable to that of non-targeting shRNA controls, demonstrating that LIMD1 is both necessary and sufficient for PD-L1 regulation at the protein level in these cells. To address potential off-target effects, we generated a lentivirus with an independent shRNA sequence that targets the LIMD1 3′UTR (shLIMD1_2 and rrLIMD1_2) and the same phenotype was observed in A549 and H1299 cells (Fig S1A).

Similarly, LIMD1 knockdown with siRNA in H1299, A549, H3122, and H358M lung carcinomas increased PD-L1 protein, particularly after interferon-gamma (IFNγ) treatment (Figs 1C and D and S1B). This phenotype was also observed in primary human small airway epithelial cells (SAECs) (Fig 1E and F) and murine cancer lines, including CT26 colorectal carcinoma and MCA-205 fibrosarcoma, where LIMD1 was overexpressed (Figs 1G and H, and S1C and D). Interestingly, depletion of Ajuba, a LIMD1 paralogue that is frequently inactivated in pleural mesothelioma models (Tanaka et al, 2015), also increased PD-L1 expression (Fig S1E), implying that members of the zyxin family share functional roles in regulating PD-L1 expression.

**Figure 2.**
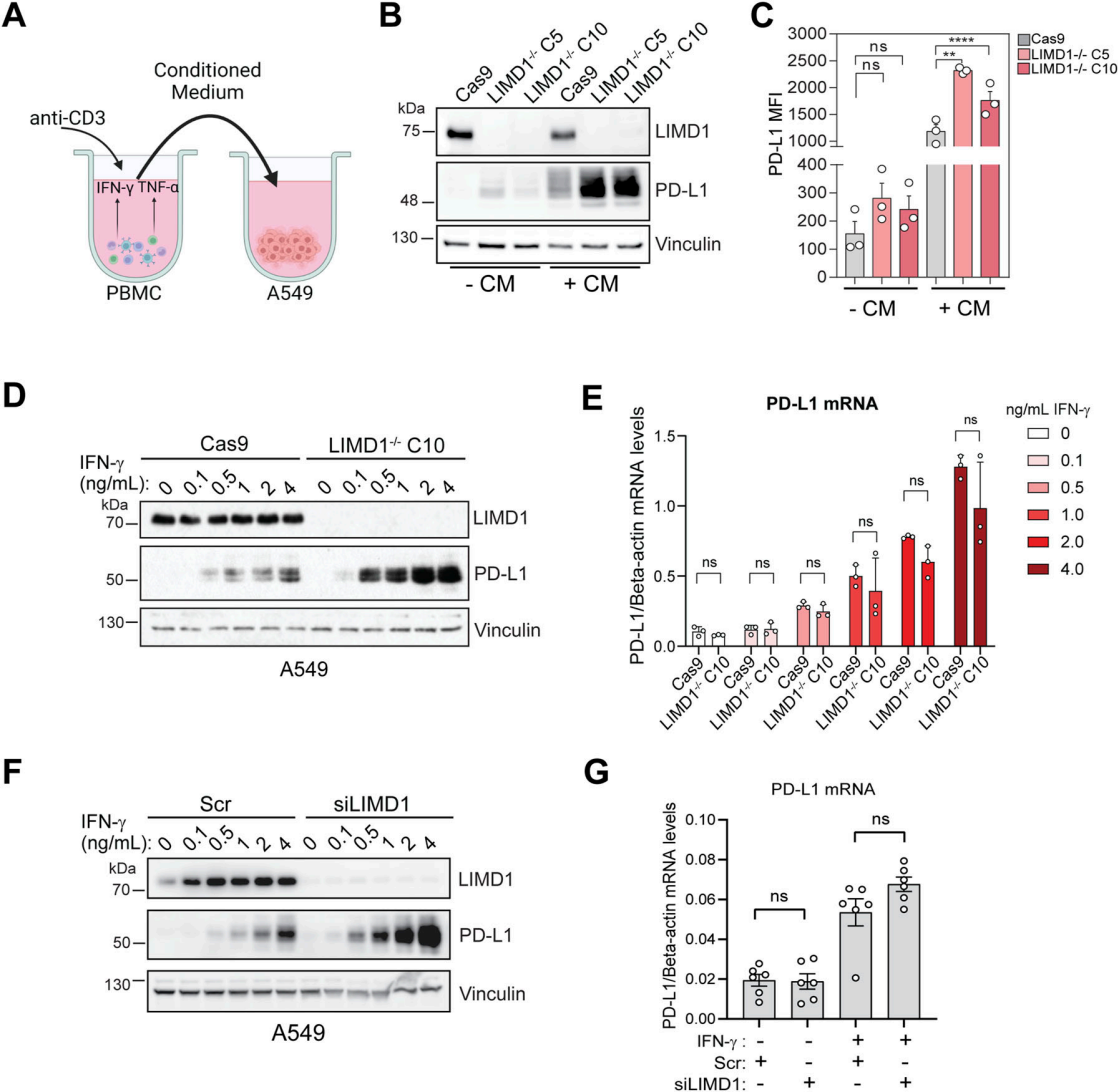
LIMD1 loss up-regulates PD-L1 protein in A549. **(A)** Schematic showing collection of CM from PBMCs activated with anti-CD3 (5 μg/ml, 3 d) for induction of PD-L1 expression in A549. Immunoblot **(B)** and flow cytometric analysis **(C)** of LIMD^+/+^ or LIMD^−/−^ A549 treated with PBMC-conditioned medium for 24 h. Data show the mean ± SEM for three biological replicates . ****P < 0.0001, **P < 0.01, *P < 0.05 from one-way ANOVA with Sidak’s multiple comparisons test. Analysis of PD-L1 protein by immunoblot **(D)** and mRNA levels by qRT-PCR **(E)** in LIMD1 knockout (LIMD1^−/−^ C10) and LIMD^+/+^ A549 after stimulation with IFNγ for 24 h. The graph shows the mean ± SEM. *CD274* mRNA was normalised to beta-actin for three independent experiments. n.s. = non-significant according to one-way ANOVA with Sidak’s multiple comparisons test. **(F)** PD-L1 immunoblot in WT A549 transfected with LIMD1 siRNA or control siRNA (Scr) and treated with IFNγ for 24 h. **(G)** qRT-PCR of PD-L1 mRNA in WT A549 transfected with Scr or LIMD1 siRNA and treated with vehicle or IFNγ (1 ng/ml) for 24 h. Data are shown as the mean ± SEM. *CD274* mRNA was normalised to beta-actin for six independent experiments. n.s. = non-significant according to the Mann–Whitney test. CM, conditioned medium. Source data are available for this figure.

**Figure S2.**
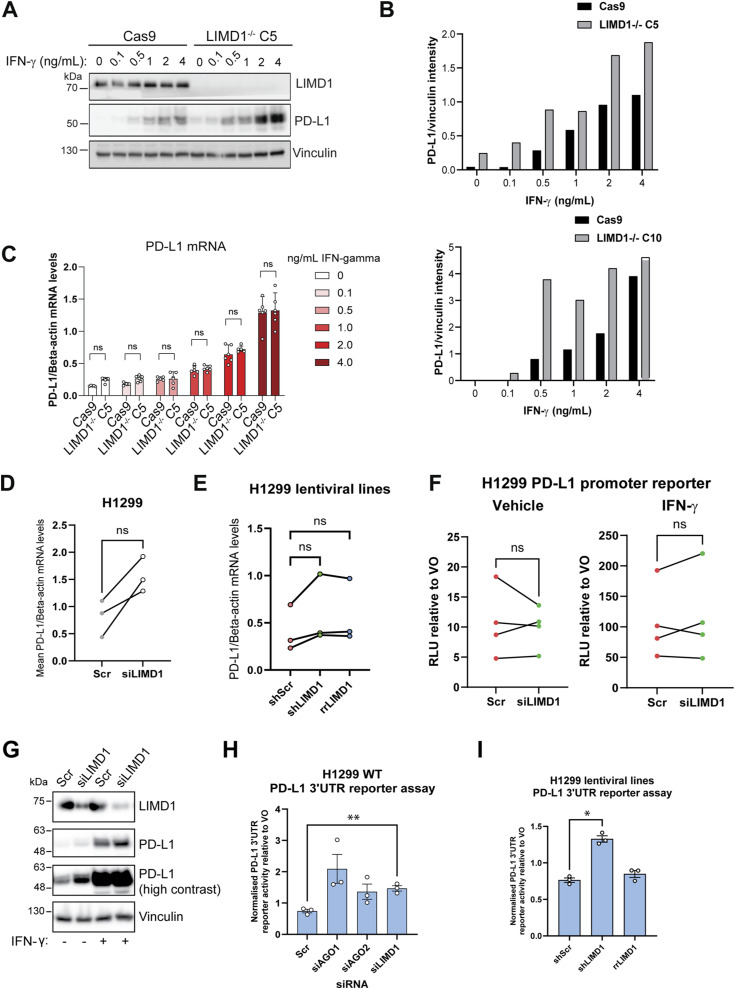
In A549 LIMD1 depletion does not upregulate PD-L1 mRNA and in H1299 cells the PD-L1 mRNA 3’ UTR is de-repressed by LIMD1 depletion. A549 Cas9 control and LIMD1^−/−^ clone 5 were treated with increasing concentrations of recombinant IFNγ overnight and then harvested for whole-cell lysates for immunoblotting **(A)** or total RNA for RT–qPCR of PD-L1 (C). Graphs in **(B)** show densitometry for PD-L1 intensity normalised to the loading control. **(C)** Graph shows mean PD-L1 mRNA levels normalised to beta-actin mRNA levels for five independent experiments. Error bars denote SEM. No statistically significant difference in PD-L1 mRNA was seen between Cas9 and LIMD1^−/−^ clone 5 according to two-way ANOVA with Sidak’s multiple comparisons test. **(D)** qPCR for PD-L1 mRNA in H1299 cells transfected with Scr or LIMD1 siRNA. The graph shows PD-L1/beta-actin mRNA levels for three independent biological repeats each consisting of three replicates. Ns = not significant according to the Wilcoxon matched-pairs signed-rank test (*P* = 0.25). **(E)** qRT-PCR for PD-L1 mRNA in H1299 lentiviral lines. The graph shows PD-L1/beta-actin mRNA levels for three independent biological repeats. Ns = not significant according to the Wilcoxon matched-pairs signed-rank test (*P* = 0.25). **(F)** PD-L1 promoter reporter assays in H1299 transfected with Scr or LIMD1 siRNA. Cells were treated with vehicle (left) or 1 ng/ml IFNγ (right) overnight before harvesting. Data show means of PD-L1 promoter NanoLuc activity normalised to firefly luciferase activity relative to empty vector (VO) for four paired independent repeats. Ns, not significant according to a paired *t* test. **(G)** Representative immunoblots for H1299 lysates from the assay for endogenous proteins (LIMD1, PD-L1, and vinculin as a loading control). **(H)** Luciferase reporter assay for the PD-L1 3′UTR in H1299 WT cells transfected with the indicated siRNAs. Data show the mean ± SEM of RLuc/FFLuc activity relative to the empty vector (VO) for three independent experiments. **P < 0.01 according to Welch’s *t* test. **(I)** Luciferase reporter assay for the PD-L1 3′UTR in H1299 lentiviral lines. Data show the mean ± SEM of RLuc/FFLuc activity relative to the empty vector (VO) for three independent experiments. *P < 0.05 according to Welch’s *t* test.

Given tumour PD-L1 expression can be regulated at multiple levels, including *CD274* transcription, mRNA translation, and protein turnover, we next defined which regulatory mechanisms are impacted by LIMD1 loss and whether this is conserved across lung cancer models. In H1299 cells, where LIMD1 depletion up-regulates PD-L1 protein, we also observed increased PD-L1 mRNA levels upon LIMD1 knockdown, but this was not statistically significant (Fig 2A). Furthermore, LIMD1 silencing did not modulate *CD274* promoter reporter activity, either with or without IFNγ treatment (Fig S2A). We have previously shown regulation of microRNA-mediated silencing by LIMD1 (James et al, 2010; Bridge et al, 2017; Crozier et al, 2026). To test the effect of LIMD1 depletion on PD-L1 silencing, we used a luciferase reporter containing the 3′UTR of *CD274*. LIMD1 depletion in H1299 cells led to derepression of this reporter in a manner similar to that seen when canonical miRNA pathway proteins AGO1 and AGO2 were silenced (Fig 2B). A similar derepression of this reporter was observed in the H1299 shLIMD1 lentiviral line, with repression rescued in the rrLIMD1 line (Fig 2C). Thus in H1299 cells, LIMD1 depletion abrogates microRNA-mediated silencing of the PD-L1 3′UTR, leading to enhanced PD-L1 protein levels.

To further probe the LIMD1-PD-L1 regulatory relationship, we generated isogenic A549 LIMD1−/− clones (C5 and C10) alongside a Cas9-expressing LIMD1+/+ control. In keeping with RNAi models, A549 LIMD1−/− cells exhibited markedly elevated PD-L1 protein after inflammatory challenge with conditioned medium from anti-CD3–activated PBMCs (Fig 2D–F). Recombinant IFNγ treatment similarly induced substantially higher PD-L1 protein in LIMD1−/− than LIMD1+/+ cells, confirmed by both immunoblotting and flow cytometry (Figs 2G and S2B). Despite this striking protein difference, *CD274* mRNA levels remained comparable between knockout and control cells (Figs 2H and S2C), indicating that LIMD1 loss can elevate PD-L1 independently of increased *CD274* transcription in this context.

**Figure. S3.**
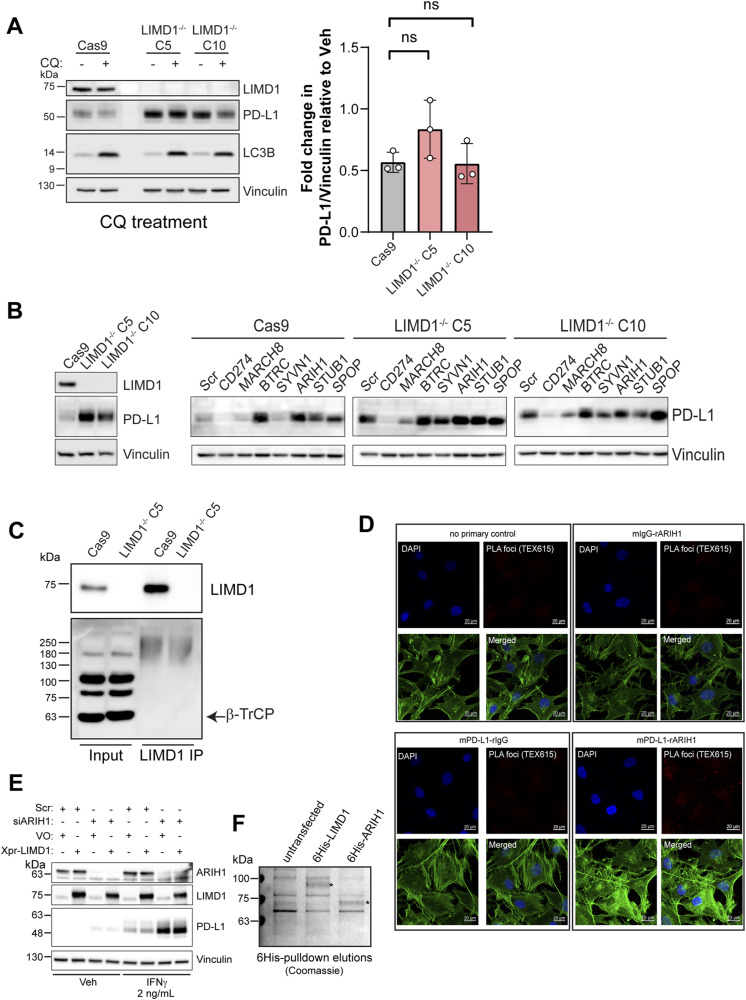
LIMD1 depletion in A549 does not affect lysosomal degradation of PD-L1. Supplemental data for siRNA screen, PLA experiments and in vitro ubiquitination assay. **(A)** A549 Cas9 and LIMD1^−/−^ cells (clone 5 and clone 10) were treated with vehicle or CQ at 40 μM overnight. Whole-cell lysates were immunoblotted for PD-L1, LIMD1, LC3B, and vinculin as a loading control. The graph shows mean fold change in PD-L1 levels relative to vehicle treatment for each line for three independent experiments. Ns means not significant according to the *t* test. **(B)** Representative Western blots for PD-L1 and vinculin for siRNA transfections in A549 Cas9 and LIMD1^−/−^ C5 and C10 clones for main Fig 3D. **(C)** LIMD1 immunoprecipitation in A549 Cas9 and LIMD1−/− C5. Inputs and IP elutions were also blotted for β-TrCP (∼ 63 kDa, arrow). **(D)** Representative images of control conditions (no primary control, mIgG-rARIH1, mPD-L1-rIgG) and test condition (mPD-L1-rARIH1) for PLA. Foci for PLA are shown in red (Texas Red 615). Cells were also stained with DAPI to highlight nuclei and phalloidin-AF488 to show actin cytoskeleton. Scale bars show 20 microns. **(E)** Immunoblots of A549 cell lysates where cells were transfected with Scr or ARIH1 siRNA for 3 d and then transfected with empty vector (VO) pcDNA4 or pcDNA4 Xpr-LIMD1. 24 h before harvesting, cells were treated with vehicle or IFNγ at 2 ng/ml. Vinculin was probed as a loading control. **(F)** Coomassie blue–stained gel for elutions from Ni^2+^ agarose pulldowns from HEK293FT cell lysates expressing the indicated 6His-tagged proteins. * denotes the band for the protein of interest in each case. Eluted proteins were subsequently used in in vitro ubiquitination assays. CQ, chloroquine.

In contrast to H1299 cells, depletion of LIMD1 in A549 did not alter the activity of the PD-L1 3′UTR luciferase reporter (Fig S3A). In contrast, knockdown of Ajuba and AGO1 did lead to derepression of this reporter gene. These results suggest that in A549 cells, the role of LIMD1 in miRNA-mediated silencing is not responsible for the increased PD-L1 protein expression observed after LIMD1 loss, but related family member proteins exhibit the capacity to post-transcriptionally regulate PD-L1 at the 3′UTR.

**Figure 3.**
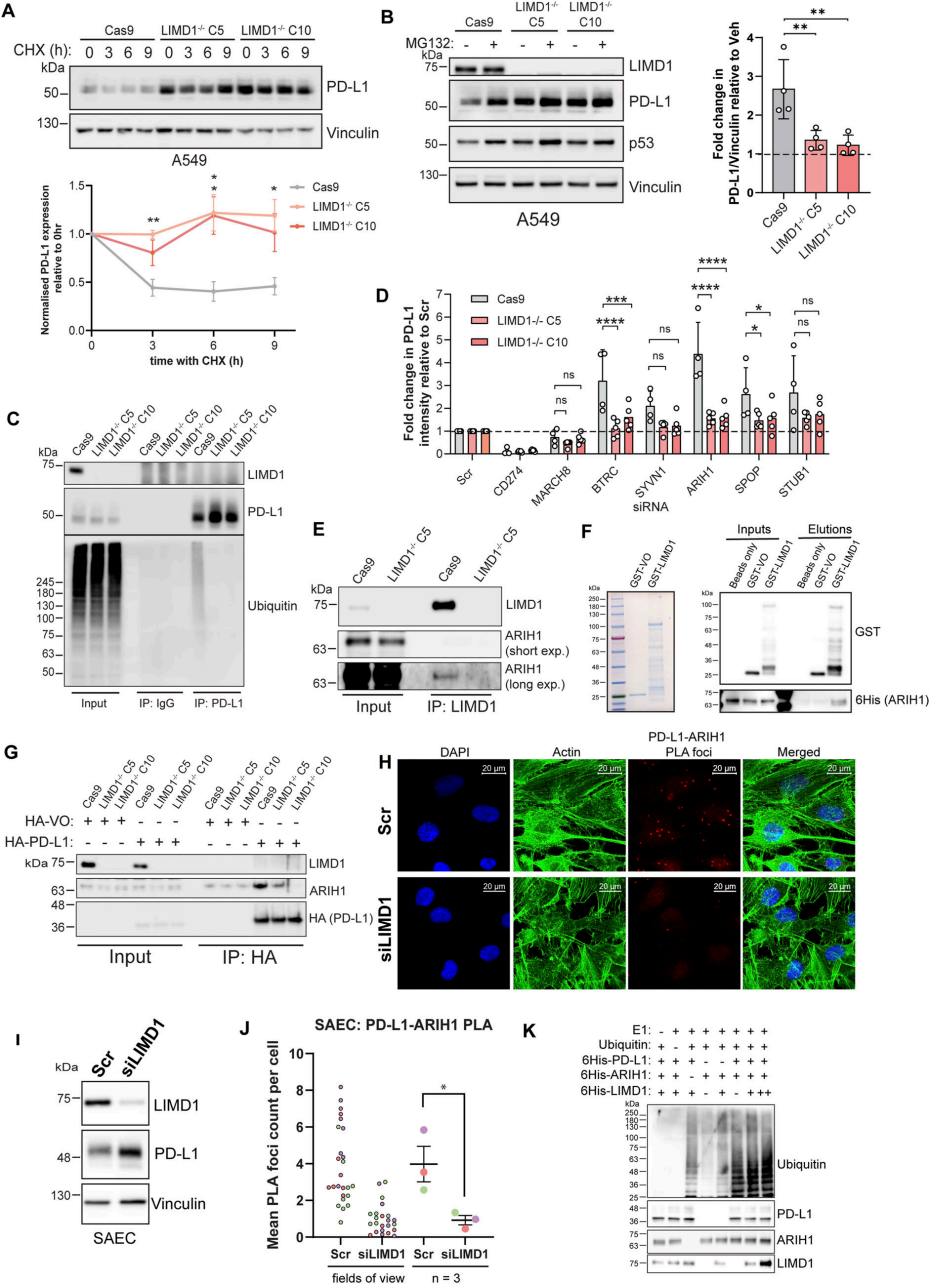
LIMD1 loss increases PD-L1 protein stability by reducing PD-L1 ubiquitination by ARIH1. **(A)** Immunoblot for the PD-L1 protein level in A549 Cas9 and LIMD1−/− cells after incubation with cycloheximide (40 μg/ml from 0 to 9 h), with vinculin probed as a loading control. The graph shows normalised quantification of PD-L1 by densitometry over cycloheximide time course. Data represent the mean ± SEM for six independent experiments.**P < 0.01, *P < 0.05 according to a paired *t* test. **(B)** Analysis of A549 LIMD1, PD-L1, and p53 levels by immunoblot after treatment with MG132 (10 μM for 24 h). The graph shows densitometry for fold change of the normalised PD-L1 protein level relative to the vehicle control condition. Data represent the mean ± SEM for four independent experiments. **P < 0.01 according to one-way ANOVA with Dunnett’s multiple comparisons test. **(C)** A549 cells were treated with IFNγ for 24 h and with 10 μM MG132 for 6 h. PD-L1 was then immunoprecipitated from whole-cell lysates, and elutions and input samples were immunoblotted for ubiquitin and PD-L1. Control IPs were performed with rabbit IgG. **(D)** Immunoblot densitometry of fold change in PD-L1 relative to Scr siRNA-transfected A549 Cas9 or LIMD1^−/−^ C5 and C10 cells upon siRNA depletion of various PD-L1 regulators or *CD274* itself. PD-L1 density was normalised to the corresponding vinculin loading control. Data show mean fold change for at least four independent experiments ± SEM. *P < 0.05, **P < 0.01, ***P < 0.001, ****P < 0.0001 according to two-way ANOVA with Dunnett’s multiple comparisons test. **(E)** Immunoblots of endogenous co-immunoprecipitation of ARIH1 with LIMD1 in A549 Cas9 and C5 LIMD1^−/−^ cells. **(F)** GST pull-down assay of 6His-ARIH1 with glutathione Sepharose beads alone, GST-VO, or GST-LIMD1. The left image shows a Coomassie blue–stained gel of purified GST-VO and GST-LIMD1. Right panels are immunoblots of GST and 6His-tag. **(G)** Co-immunoprecipitation of HA-PD-L1 in A549 Cas9 and LIMD1^−/−^ cells with ARIH1. **(H)** Representative images of SAEC transfected with Scr or LIMD1 siRNA for PLA between PD-L1 and ARIH1. Cells were also stained with DAPI to show nuclei and phalloidin-AF488 to highlight F-actin. **(I)** Representative immunoblot for SAEC transfected with Scr or siLIMD1 to show LIMD1 knockdown and PD-L1 up-regulation. Vinculin was probed as a loading control. **(J)** Quantification of PLA foci per cell. The graph shows results for each field imaged (left) and mean data for three independent biological repeats (right). *P < 0.05 according to one-tailed Welch’s *t* test. **(K)** In vitro ubiquitination assay. 6His-tagged PD-L1, ARIH1, and LIMD1 were purified from HEK293FT cells separately. These were combined in reactions as indicated with E1, E2 (UBCH7), and ubiquitin and incubated for 4 h at 37°C. Reactions were quenched with 2× non-reducing loading dye and subjected to immunoblotting for the indicated proteins. Source data are available for this figure.

In the light of the data in A549 showing lack of transcriptional and post-transcriptional changes in PD-L1 after LIMD1 loss, we next tested whether the stability of PD-L1 protein was altered. Using a cycloheximide time course to inhibit translation, we found significant stabilisation of PD-L1 protein in A549 LIMD1^−/−^ cells (Fig 3A). We next investigated specific degradation pathways that may be involved, namely, lysosomal and proteasomal mechanisms. After inhibition of lysosomal degradation with chloroquine, we observed LC3B enrichment and a consistent decrease in the PD-L1 level in LIMD1^+/+^ and LIMD1^−/−^ cells (Fig S3B), implying lysosomal degradation of PD-L1 is unchanged by LIMD1 loss.

Assessment of proteasome function using the inhibitor MG132 revealed significant PD-L1 enrichment in A549 LIMD1^+/+^ only, suggesting impaired proteasomal degradation of PD-L1 in LIMD1-deficient cells (Fig 3B).

### ARIH1 mediates PD-L1 ubiquitination in a LIMD1-dependent manner

Consistent with the proteasome inhibition data, immunoprecipitation of PD-L1 revealed reduced polyubiquitination in A549 LIMD1^−/−^ (Fig 3C), indicating that the high PD-L1 expression level characteristic of LIMD1-deficient cells is, at least partially, a result of impaired PD-L1 ubiquitination and proteasomal turnover. Recent studies have identified several regulators involved in the ubiquitination of PD-L1 (reviewed in Yamaguchi et al, 2022). To identify E3 ubiquitin ligases that may differentially regulate PD-L1 depending on the LIMD1 status, we used a candidate approach to silence known regulators and measure the PD-L1 level in A549 cells (Figs 3D and S3C). Interestingly, we observed a marked and selective relative increase in PD-L1 in A549 LIMD1^+/+^ over LIMD1^−/−^ clones after depletion of the E3 ligases BTRC (βTrCP) or ARIH1, which have been shown to mediate PD-L1 ubiquitination (Li et al, 2016; Wu et al, 2021; Xie et al, 2025). Conversely, depletion of other E3 ligases, including MARCH8, SYVN1, and STUB1, did not modulate PD-L1 differentially between LIMD1^−/−^ and control cells. Given the precedent of LIMD1 interacting with E3 ubiquitin ligases (e.g., VHL [Foxler et al, 2012]), we confirmed that LIMD1 interacts with ARIH1 by endogenous co-immunoprecipitation in A549 (Fig 3E). Furthermore, with a GST pull-down assay of purified proteins, we found a 6His-tagged ARIH1 could directly interact with GST-LIMD1 (Fig 3F). However, no interaction was found between LIMD1 and BTrCP in A549 cells (Fig S3D). Closer examination of molecular interactions revealed that co-immunoprecipitation of HA-PD-L1 with ARIH1 was reduced in A549 LIMD1^−/−^ cells (Fig 3G). Using the proximity ligation assay (PLA), we found that LIMD1 depletion with siRNA in SAECs also reduced the interaction between PD-L1 and ARIH1, and this was accompanied by increased PD-L1 protein expression (Figs 3H–J and S3E). These findings reveal a previously unrecognised role for LIMD1 in facilitating the interaction between PD-L1 and ARIH1, suggesting that LIMD1 may generally enhance the functionality of E3 ubiquitin ligases in regulating protein turnover. In the absence of LIMD1, ARIH1-dependent PD-L1 ubiquitination and degradation are impaired, and PD-L1 protein accumulates. The overexpression of LIMD1 in cells where ARIH1 was depleted with siRNA did not change PD-L1 protein expression, indicating ARIH1 is required for LIMD1-dependent PD-L1 regulation (Fig S3F).

Using purified proteins (see the Materials and Methods section on *6His-tagged protein purification*), we reconstituted a minimal ubiquitination reaction containing E1, E2, ubiquitin, 6His-ARIH1, and 6His-PD-L1 to directly test whether 6His-LIMD1 can potentiate ARIH1-mediated PD-L1 ubiquitination (Figs 3K and S3G). Under these conditions, ARIH1 supported PD-L1 ubiquitination, and inclusion of recombinant LIMD1 increased the abundance of higher molecular weight ubiquitinated PD-L1 species, consistent with LIMD1 enhancing the efficiency of the ARIH1→PD-L1 ubiquitination step. Together with the cellular co-IP/PLA data, these findings support a model in which LIMD1 acts as a factor that promotes productive ARIH1-PD-L1 engagement and thereby facilitates ubiquitin-dependent proteasomal turnover of PD-L1.

### LIMD1 loss increases PD-L1–dependent suppression of human T-cell activation

Having established that LIMD1 restrains PD-L1 abundance through post-transcriptional and post-translational mechanisms, we next asked whether this translates into altered tumour–immune interactions. We therefore used human PBMC co-culture systems to directly test the functional consequences of tumour cell LIMD1 loss on T-cell activation and on sensitivity to PD-1/PD-L1 blockade.

**Figure 4.**
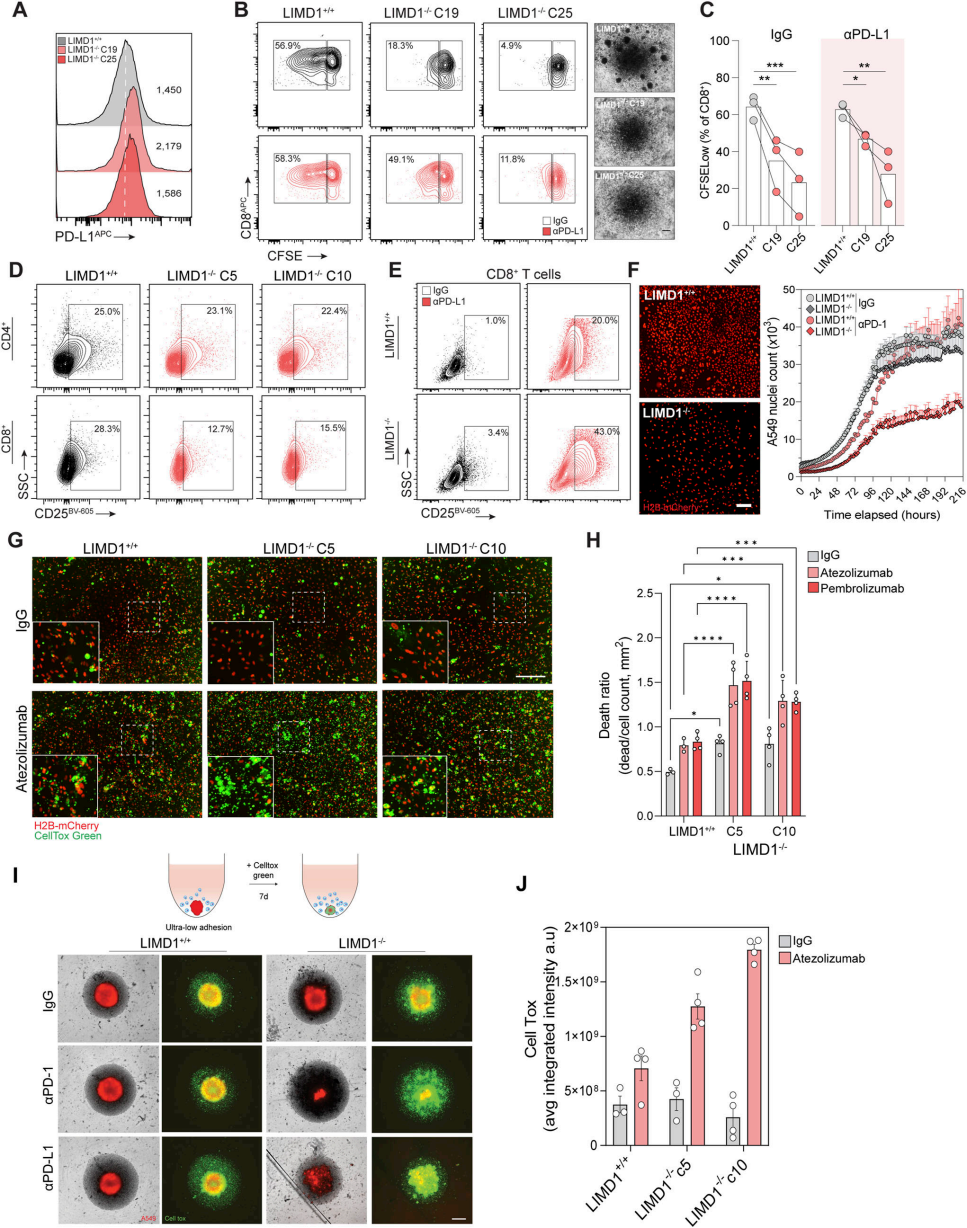
LIMD1-deficient cells suppress T-cell activation and are sensitive to PD-L1 blockade in vitro. **(A)** Surface PD-L1 expression in H1299 cells measured by flow cytometry. Values indicate the median fluorescence intensity. **(B)** CD8^+^ T-cell proliferation in co-culture with H1299 target cells with or without anti-PD-L1 (10 μg/ml). Phase-contrast images show representative cultures after 5 d (scale = 100 μm). **(C)** CD8^+^ T-cell proliferation within H1299 co-cultures. ***P < 0.001, **P < 0.01, *P < 0.05 for two-way ANOVA with the Holm–Sidak correction. Data represent PBMC from n = 3 independent healthy donors. **(D)** Flow cytometric measurement of CD25 expression in T cells after 5-d co-culture with A549 (untreated). **(E)** Measurement of CD8^+^ T-cell proliferation within PBMC-A549 co-cultures after ICB with atezolizumab (10 μg/ml). **(F)** Cell proliferation curves showing A549 growth within PBMC co-cultures after ICB with atezolizumab. Representative endpoint images show A549 nucleus density at 200 h (scale = 300 μm). **(G, H)** Live-cell microscopy measurement of cytotoxicity within A549-PBMC 2D co-cultures treated with atezolizumab or IgG control (10 μg/ml). Representative endpoint images show nucleus density and cell death signal at 150 h (scale bar = 150 μm). Data are the mean ± SEM, n = 4 (****P < 0.0001, ***P < 0.001, *P < 0.05, two-way ANOVA with the Holm–Sidak correction). **(I, J)** Microscopy-based measurement of cytotoxicity within atezolizumab-treated A549-PBMC 3D spheroid co-cultures. **(I)** Representative endpoint images show nucleus density at 175 h (scale bar = 250 μm). **(J)** Data are the mean ± SEM for a representative PBMC donor. PBMCs were pre-activated with anti-CD3 (2 μg/ml for 3 d), and cultures were treated with IgG control, pembrolizumab, or atezolizumab (10 μg/ml for 7 d).

Consistent with the biochemical phenotype, LIMD1-deficient H1299 cells displayed increased surface PD-L1 (Fig 4A) and more strongly suppressed activated CD8^+^ T-cell proliferation than control cells (Fig 4B and C). In an allogeneic A549-PBMC co-culture model, LIMD1 loss similarly reduced T-cell activation, evidenced by lower CD25 induction on both CD4^+^ and CD8^+^ compartments (Fig 4D). Notably, PD-L1 blockade with atezolizumab preferentially rescued activation in LIMD1-deficient cultures, with a larger increase in CD8^+^ CD25 expression relative to LIMD1+/+ co-cultures (Fig 4E), supporting the interpretation that LIMD1 loss enhances functional PD-L1 checkpoint activity in vitro. We note that the differing basal levels of CD25 on CD8^+^ T cells in Fig 4D and E are likely due to different donors for PBMCs.

To test whether this altered checkpoint dependence impacts tumour cell fitness in the presence of immune effector cells, we next tracked A549 reporter cells that were fluorescent during long-term PBMC co-culture. In the presence of IgG control, LIMD1+/+ and LIMD1−/− tumour cells grew at similar rates; however, blocking PD-1 with pembrolizumab specifically slowed the growth of LIMD1^−/−^ cells (Fig 4F). In parallel, cytotoxicity dye incorporation revealed increased tumour cell apoptosis after checkpoint blockade, with a stronger effect in LIMD1-deficient co-cultures (Fig 4G and H). We note that LIMD1^−/−^ tumour cells showed a modestly increased death signal compared with LIMD1^+/+^ cells even in IgG-treated co-cultures. Because these cultures contained activated PBMCs, this condition does not measure spontaneous tumour cell death in isolation. The increased death signal may therefore reflect basal immune-mediated killing and/or altered tumour cell fitness associated with LIMD1 loss. Importantly, PD-L1 blockade further increased tumour cell death preferentially in LIMD1^−/−^ co-cultures, supporting the conclusion that elevated PD-L1 functionally restrains antitumour immune activity in LIMD1-deficient cells.

This trend was also seen in 3D spheroid co-cultures, where LIMD1−/− spheroids showed more cell death compared with LIMD1+/+ controls after blocking (Fig 4I and J). These findings indicate that LIMD1 loss increases functional PD-L1 checkpoint activity and creates enhanced dependence on the PD-1/PD-L1 axis in tumour–PBMC co-cultures.

### Clonal LIMD1 loss associates with elevated tumour PD-L1 in LUAD and with ICI response

**Figure 5.**
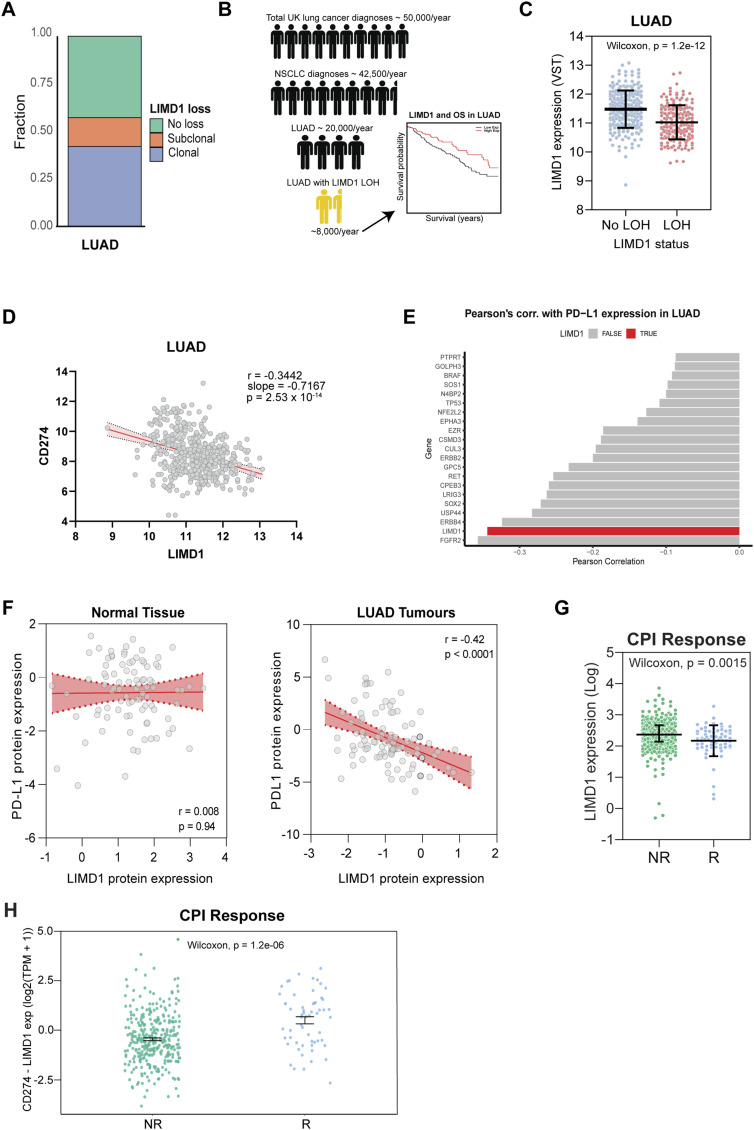
Clonal LIMD1 LOH is prevalent in LUAD and correlated with PD-L1 expression. **(A)** Proportion of samples in the TRACERx 421 cohort exhibiting clonal or subclonal LOH for LIMD1 in LUAD. **(B)** Estimates of numbers of lung cancer patients in the UK affected by LIMD1 LOH. One “stick man” figure is equivalent to 5,000 patients. **(C)** LIMD1 mRNA expression in LUAD samples exhibiting LOH or no LOH for LIMD1. The line shows median LIMD1 expression ± the interquartile range. *P*-values were calculated according to the Wilcoxon signed-rank test. **(D)** Correlation analysis of LIMD1 and PD-L1 mRNA expression from patients from the TRACERx-421 cohort. **(E)** Pearson’s correlations between expression of PD-L1 and 20 most-correlated COSMIC lung cancer drivers and LIMD1, sorted by the magnitude of correlation. **(F)** Proteomic analysis quantifying LIMD1 levels and PD-L1 levels in LUAD and matched healthy adjacent tissue (n = 100). Data are obtained from the Clinical Proteomic Tumor Analysis Consortium’s dataset of lung adenocarcinomas. For D, E, lines of best fit were determined with linear regression analysis and red shaded areas denote the 95% confidence intervals. **(G)** LIMD1 mRNA expression (log-transformed) in NSCLC patients, taken from the Patil et al, and Banchereau et al cohorts, stratified by response to ICB therapy. *P*-value was calculated according to the Wilcoxon signed-rank test. **(H)** Metric to combine LIMD1 and *CD274* expression in the Patil et al and Banchereau et al cohorts was calculated for each patient (*CD274*-LIMD1 exp (log2 (TPM+1))) and stratified by response to ICI. *P*-value was calculated according to the Wilcoxon signed-rank test.

LIMD1 resides at chromosome 3p21.3, a region frequently affected by early copy-number loss in lung cancer (Sharp et al, 2008). We therefore examined the TRACERx-421 cohort of multiregion-sampled NSCLC (Bailey et al, 2021; Frankell et al, 2023) to define the prevalence and timing of *LIMD1* loss of heterozygosity (LOH) and to relate LIMD1 status to immune checkpoint expression. In LUAD, LIMD1 LOH was common and frequently clonal (∼40%) (Fig 5A), which is consistent with LIMD1 loss occurring early during tumour evolution. Based on these frequencies, a conservative extrapolation suggests that LIMD1 LOH could affect several thousand LUAD cases annually in the UK (Fig 5B). Moreover, in the recently published allele-specific phylogenetic analysis of copy-number alterations algorithm (Pawlik et al, 2025), it was observed that Chr 3p LOH is truncal in most of the LUAD samples in the TRACERx-421 cohort, again showing that LIMD1 LOH is an early event during tumour evolution.

As expected, LUAD tumours with *LIMD1* LOH exhibited lower LIMD1 mRNA expression (Fig 5C). Across LUAD, LIMD1 mRNA expression inversely correlated with *CD274* mRNA (Fig 5D), and *LIMD1* ranked among the strongest inverse correlates of *CD274* within a panel of recurrent NSCLC driver genes (Fig 5E). Extending this to the protein level, analysis of the LUAD dataset from the Clinical Proteomic Tumor Analysis Consortium (Gillette et al, 2020) revealed a significant inverse relationship between LIMD1 and PD-L1 protein in tumours, a trend not observed in matched normal adjacent tissue (Fig 5F). Importantly, because tumour-level *CD274* mRNA is strongly influenced by inflammatory signalling and tumour cellular composition, transcriptional associations in patient cohorts can reflect both microenvironmental cues and tumour-intrinsic regulation. Our isogenic A549 models demonstrate that LIMD1 loss can markedly increase PD-L1 protein without a commensurate increase in *CD274* mRNA, supporting a tumour-intrinsic post-transcriptional/post-translational mechanism that can coexist with, and potentially amplify, transcriptional PD-L1 induction in vivo.

**Figure S4.**
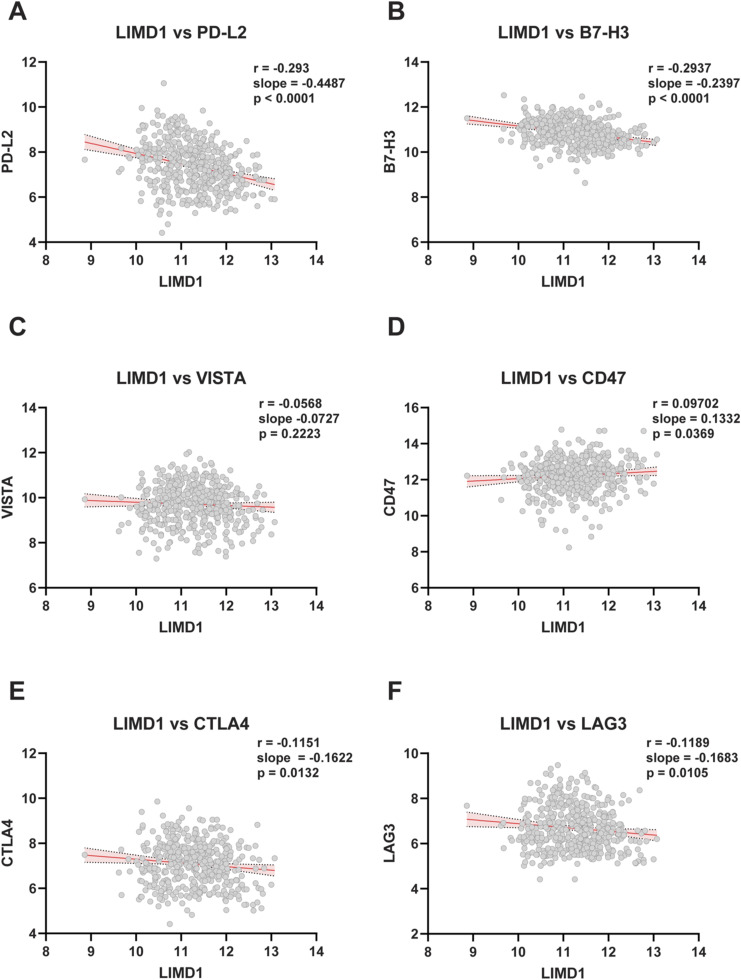
Correlation analyses of LIMD1 mRNA with other immune checkpoint genes in the TRACERx LUAD cohort. **(A, B, C, D, E, F)** Correlation analyses of mRNA expression for LIMD1 and indicated immune checkpoint genes for LUAD samples in the TRACERx-421 cohort. For each analysis, Pearson’s correlation coefficients (r) and *P*-values (two-tailed) were calculated. The red shaded areas show the 95% confidence intervals.

To assess whether LIMD1 status relates more broadly to immune checkpoint programmes, we in addition tested associations between LIMD1 expression and other checkpoint genes in TRACERx LUAD samples. LIMD1 showed significant inverse correlations with multiple checkpoint transcripts (including PDCD1LG2/PD-L2 and CD276/B7-H3), whereas other checkpoints showed weaker or non-significant relationships (Fig S4A–F). Notably, among the checkpoints tested, the LIMD1-*CD274* relationship was the strongest inverse association (r = −0.344).

Finally, to evaluate potential clinical relevance for immunotherapy stratification, we examined independent ICI-treated NSCLC patient cohorts with pre-treatment RNA sequencing (Banchereau et al, 2021; Patil et al, 2022). Patients who responded to ICIs exhibited significantly lower LIMD1 expression than non-responders (Fig 5G). Moreover, a simple combined metric capturing *CD274* expression and the inverse of LIMD1 expression (*CD274* - LIMD1 (log2(TPM+1))) (where a high number indicates *high* PD-L1 expression combined with *low* LIMD1 expression) distinguishes responders from non-responders (Fig 5H), consistent with response enrichment in tumours with higher PD-L1 and lower LIMD1 expression. Together, these analyses link a common, often clonal tumour suppressor loss event to elevated PD-L1 and to immunotherapy response patterns across cohorts.

In summary, these results support LIMD1 as a multilayered negative regulator of PD-L1. In H1299 cells, LIMD1 depletion derepresses the *CD274* 3′UTR in a manner consistent with impaired miRNA-mediated silencing, whereas in A549 and primary airway epithelial models, LIMD1 loss stabilises PD-L1 protein by disrupting ARIH1-dependent ubiquitination and proteasomal turnover. Functionally, LIMD1-deficient tumour cells suppress human T-cell activation in vitro and are preferentially sensitised to PD-1/PD-L1 blockade. Clinically, LIMD1 LOH is common and often clonal in NSCLC, and associates with increased tumour PD-L1 in LUAD, and low LIMD1 expression is enriched in ICI responders across independent cohorts—together supporting LIMD1 status as a candidate biomarker to refine immunotherapy stratification.

## Discussion

Immune evasion through up-regulation of the immune checkpoint ligand PD-L1 is a central mechanism by which tumours escape cytotoxic T-cell surveillance and remains a major determinant of response to ICB in NSCLC. Although transcriptional pathways regulating PD-L1 expression, such as interferon-dependent JAK-STAT signalling (Garcia-Diaz et al, 2017) and hypoxia-driven HIF-1α activation (Barsoum et al, 2014; Noman et al, 2014), have been extensively characterised, the tumour-intrinsic mechanisms that control PD-L1 protein stability remain less well defined.

Here, we identify the tumour suppressor LIMD1 as a previously unrecognised regulator of PD-L1 abundance. We show that LIMD1 constrains PD-L1 through two mechanisms: by supporting microRNA-mediated repression of the *CD274* 3′UTR and by promoting ARIH1-dependent ubiquitination and proteasomal turnover of PD-L1 protein. Loss of LIMD1 therefore leads to accumulation of PD-L1 and increased immune checkpoint activity.

Functionally, LIMD1-deficient tumour cells suppress T-cell activation in vitro and display increased dependence on PD-1/PD-L1 signalling. A limitation of this study is that basal proliferation and spontaneous cell death of LIMD1^+/+^ and LIMD1^−/−^ cells were not directly quantified under all co-culture conditions. Previous work demonstrated that LIMD1 interacts with pRB, represses E2F-dependent transcription, and suppresses tumour growth, consistent with a role for LIMD1 in regulating cell-cycle progression and tumour cell fitness (Sharp et al, 2004). Therefore, intrinsic differences in proliferation or survival may contribute to the absolute tumour cell death signals observed in co-culture. Nevertheless, because the increased death was measured in the presence of activated PBMCs and was further enhanced by PD-L1 blockade, the data remain consistent with a model in which LIMD1 loss promotes PD-L1–dependent immune suppression while leaving tumour cells susceptible to immune-mediated killing when this checkpoint is inhibited.

In human tumours, LIMD1 loss is frequently clonal in lung adenocarcinoma and is associated with increased PD-L1 expression and enrichment among patients responding to ICB.

These findings position LIMD1 as a tumour-intrinsic regulator of immune checkpoint biology. Rather than directly driving tumorigenesis along with other genetic ablations, loss of *LIMD1* appears to shape the immune interface of tumour cells by stabilising PD-L1 and thereby enhancing immune evasion. This provides a mechanistic framework linking an early tumour-associated genomic alteration to immune checkpoint regulation and suggests that LIMD1 status may help refine biomarker strategies for immunotherapy stratification.

Our data demonstrate that LIMD1 loss is a frequent and clonal event, with ∼40% of lung adenocarcinoma cases exhibiting LOH. This clonal nature highlights *LIMD1* loss as an early driver of tumour evolution, distinguishing it from other PD-L1 regulators that are often up-regulated later in tumour progression. Previous studies have linked truncal mutations in tumour suppressors like LKB1, PTEN, and TP53 to PD-L1 regulation via transcriptional pathways (Xu et al, 2014; Koyama et al, 2016a; Cortez et al, 2016; Peng et al, 2016).

Recent long-read sequencing studies in other epithelial cancers further suggest that the full spectrum of LIMD1 disruption may not yet be captured by conventional short-read NSCLC datasets. In colorectal cancer, ultra–low-input HiFi sequencing identified a progressive tandem repeat expansion within the 5′UTR of LIMD1 that was associated with reduced gene expression and tumour evolution, revealing a non-coding structural mechanism of silencing not detectable through standard mutation or copy-number pipelines (Wang et al, 2025 *Preprint*). Similarly, repeat-length variation at the LIMD1 locus has been linked to gene inactivation and adverse clinical outcome in head and neck squamous cell carcinoma (Handoko et al, 2025). Although such alterations have not yet been systematically evaluated in NSCLC, these findings raise the possibility that structurally complex or regulatory *LIMD1* lesions may be under-recognised in lung cancer cohorts. This is particularly relevant to the present study, as our data demonstrate that even partial or clonal LIMD1 loss is sufficient to stabilise PD-L1 protein through impaired ARIH1-dependent ubiquitination. Expanded application of long-read sequencing in NSCLC may therefore refine our understanding of how early *LIMD1* disruption contributes to tumour-intrinsic PD-L1 regulation and immune checkpoint dependence, without presupposing additional mechanisms beyond those functionally demonstrated here.

Consistent with the possibility that diverse modes of *LIMD1* disruption converge on checkpoint regulation, our previous work shows that LIMD1 binds core miRISC components AGOs and TNRC6A (Das Thakur et al, 2010; Bridge et al, 2017) and that LIMD1 depletion can weaken miRNA-dependent repression of reporters bearing artificial or native 3′UTRs. In H1299 cells, the PD-L1 (*CD274*) 3′UTR reporter is repressed in an AGO1-dependent manner, consistent with regulation by endogenously expressed miRNAs with sites in this 3′UTR (e.g., miR-155 [Yee et al, 2017]). LIMD1 depletion derepressed the PD-L1 3′UTR reporter, indicating that, at least in this context, LIMD1 limits PD-L1 via its role in miRNA-mediated silencing.

As miRNA silencing often reduces target mRNA abundance (Guo et al, 2010), the modest (non-significant) increase in PD-L1 mRNA we observe in H1299 cells after LIMD1 depletion raises the possibility that the inverse *LIMD1-CD274* mRNA relationship in LUAD reflects differential miRNA-mediated silencing in LIMD1-low versus LIMD1-high tumours. Notably, the inverse association is stronger at the protein level in Clinical Proteomic Tumor Analysis Consortium (r = −0.42) than at the mRNA level (r = −0.34). Although these are different patient cohorts, this pattern supports the practical hypothesis that PD-L1 protein (rather than *CD274* mRNA) may better capture LIMD1-linked biology and could potentially be more informative for predicting immunotherapy response.

A further layer is that LIMD1 loss can also increase PD-L1 protein stability/turnover, as seen in A549 cells, where LIMD1 loss elevates PD-L1 protein without increasing PD-L1 mRNA or derepressing the PD-L1 3′UTR reporter. Finally, *LIMD1* mRNA inversely correlates with other checkpoint genes (PD-L2 and B7-H3) in TRACERx, suggesting LIMD1 loss may contribute to coordinated dysregulation of multiple immune checkpoints. A focused and testable next step is therefore to determine whether LIMD1 depletion broadly disrupts miRNA-mediated silencing of *CD274, PDCD1LG2,* and *CD276* in LUAD-relevant contexts. Together, these observations suggest that LIMD1 loss does not simply alter tumour cell regulatory pathways in isolation but can reshape tumour–immune interactions by increasing the abundance of immune checkpoint proteins.

We have shown that LIMD1 loss in A549 cells increases PD-L1 protein stability. Unlike previous reports of chloroquine-induced PD-L1 enrichment in gastric cancer cells (Wang et al, 2019), our findings indicate that PD-L1 does not undergo significant lysosomal degradation in A549. Instead, we found that proteasomal degradation of PD-L1 is reduced with LIMD1 loss. In keeping with this, we found greater levels of polyubiquitinated PD-L1 in LIMD1-expressing A549 cells, suggesting that LIMD1 loss impairs ubiquitin-dependent proteasomal degradation of PD-L1. Previous work on LIMD1 has shown that its LIM domains mediate protein–protein interactions. During the regulation of HIF-1α, LIMD1 interacts via its LIM domains with VHL, the E3 ligase that ubiquitinates HIF-1α (Foxler et al, 2012). We now show a specific interaction between LIMD1 and the E3 ligase ARIH1, and this finding is consistent with recent studies showing that ARIH1 regulates PD-L1 stability through the ubiquitin–proteasome system (Wu et al, 2021; Xie et al, 2025). Although Liu et al highlighted ARIH1’s role in regulating PD-L1 after chemotherapy (Liu et al, 2023), our work demonstrates that LIMD1 is essential for ARIH1 function under basal conditions in the absence of inflammatory stimuli or chemotherapy. Specifically, LIMD1 interacts with ARIH1 to facilitate PD-L1 ubiquitination and subsequent proteasomal degradation. When LIMD1 is absent, the ARIH1-PD-L1 interaction is disrupted, resulting in reduced polyubiquitination and impaired degradation of PD-L1, thereby increasing its steady-state levels. This novel regulatory mechanism highlights LIMD1 as a critical modulator of PD-L1 expression and underscores its potential role in tumour immune evasion.

As PD-L1/*CD274* transcription is exquisitely sensitive to IFNγ signalling (and other inflammatory stimuli), in non-inflammatory conditions, LIMD1-dependent PD-L1 protein turnover may be critical for minimising PD-L1 protein levels that may arise from low-level transcription. In this context, LIMD1 loss and consequent impairment of PD-L1 ubiquitination and/or proteasomal degradation may lead to a steady accumulation of PD-L1 over time. With IFNγ treatment, we and others have shown that transcription of *CD274* increases dramatically. Notably, in A549 cells, we found LIMD1 loss did not affect *CD274* transcript levels and yet protein levels of PD-L1 were significantly higher in LIMD1^−/−^ cells. Thus, loss of LIMD1 in both non-inflammatory and inflammatory contexts may up-regulate PD-L1 protein levels, which facilitate tumour immune evasion. Furthermore, although ARIH1 seems to be the primary E3 ligase regulating PD-L1 in A549 cells, LIMD1 may interact with other E3 ligases in different tissue types or under different stress conditions, a hypothesis that warrants further investigation.

Clinically, PD-L1 expression has long been considered the primary biomarker for predicting response to ICIs, such as pembrolizumab and nivolumab, particularly in NSCLC. However, although PD-L1 testing remains a widely used predictor in clinical practice, its utility is limited because of the dynamic and heterogeneous nature of PD-L1 expression within tumours, leading to variability in response rates. Emerging evidence suggests that other biomarkers, such as tumour mutational burden, tumour-infiltrating lymphocytes, and specific genomic signatures, may provide more robust or complementary predictive value for ICI response (Hellmann et al, 2018; Holder et al, 2024). Despite these advancements, PD-L1 expression remains a key factor in clinical decision-making, and our findings that LIMD1 loss drives PD-L1 stabilisation offer important insights into immune checkpoint regulation, particularly in a subset of NSCLC patients. That said, more work is needed to understand this biology completely, as the range of LIMD1 expression in non-responders significantly overlaps with that of responders, preventing LIMD1 loss alone from being classified as a definitive biomarker. This overlap may reflect the multifactorial nature of ICI response, where PD-L1 stabilisation alone is insufficient to predict outcomes. Factors such as tumour mutational burden, CD8^+^ T-cell infiltration, and the broader tumour immune microenvironment likely interact with LIMD1 loss to influence immunotherapy sensitivity. Nevertheless, the enrichment of low LIMD1 expression in responders suggests that LIMD1 loss contributes meaningfully to immune checkpoint regulation and highlights the need to consider LIMD1 status as part of a broader predictive framework for ICIs.

Our study advances current understanding of immune checkpoint regulation by revealing a post-translational mechanism involving LIMD1 and ARIH1. The clinical relevance of this mechanism lies in its potential to stratify patients for immunotherapy and to guide the development of combination therapies. For example, targeting PD-L1 stabilisation pathways in LIMD1-deficient tumours may enhance the efficacy of ICIs, particularly in patients with intrinsic resistance. Furthermore, our previous work demonstrating proof-of-concept strategies for targeting LIMD1-deficient cells (Davidson et al, 2021; Saha et al, 2025 *Preprint*) raises the exciting possibility of combining LIMD1-targeted therapies with ICIs to improve outcomes in NSCLC.

A key question to address in the future is why different cellular models appear to rely differentially on LIMD1-dependent miRNA-mediated repression versus ARIH1-dependent PD-L1 ubiquitination and turnover. One possibility is that *CD274*-targeting miRNAs are expressed at different levels between models, whereas LIMD1-dependent ubiquitination may be influenced by upstream signalling pathways that regulate PD-L1 post-translational state, such as phosphorylation at S279/S283 by GSK3α.

Although in vivo studies are often used to extend mechanistic findings into physiological settings, the present work is primarily grounded in human tumour biology and tumour-intrinsic regulatory mechanisms, integrating isogenic cellular systems with large, well-annotated patient cohorts. In this context, additional in vivo validation, although informative, is not essential to support the central conclusions of this study. Indeed, it is increasingly recognised that many preclinical models, particularly syngeneic and genetically engineered mouse models, do not fully recapitulate the complexity of human tumour–immune interactions, and that findings in such systems often translate imperfectly to clinical settings (van der Worp et al, 2010; Seok et al, 2013; Mak et al, 2014). These limitations are especially relevant for immune checkpoint biology, where species-specific differences in immune regulation and tumour evolution can significantly influence outcomes. We therefore view in vivo studies as an important future direction to explore context-dependent effects, rather than a prerequisite for establishing the tumour-intrinsic mechanism described here. Ultimately, such studies may either support or refine our findings in human disease, but the present work provides a distinct and complementary contribution by defining how tumour suppressor loss directly regulates PD-L1 stability within human-relevant systems.

In summary, our study establishes LIMD1 as a novel regulator of PD-L1 stability through its interaction with ARIH1, identifying a post-translational mechanism of immune evasion in NSCLC. By demonstrating that LIMD1 loss drives PD-L1 stabilisation and influences ICI response, we provide new insights into the molecular determinants of immune checkpoint regulation. These findings have significant implications for patient stratification and the development of targeted combination therapies, offering a new avenue for improving immunotherapy outcomes in LIMD1-deficient tumours.

## Materials and Methods

### Cell culture

A549 and HEK293FT cells were cultured in DMEM (Gibco) supplemented with 10% vol/vol FCS (Gibco) and penicillin/streptomycin. H1299, H358M, H3122, H2591, H28, MCA-205, CT26, and RCC48 cells were cultured in RPMI 1640 medium (Gibco) supplemented with 10% vol/vol FCS (Gibco) and penicillin/streptomycin. SAECs were cultured in SAEC growth medium (PromoCell) with the designated supplements. Cells were cultured in humidified incubators at 37°C and 5% CO_2_. Recombinant IFNγ was purchased from Millipore (IF002).

### CRISPR/Cas9 gene editing

A549, H1299 LIMD1 knockout cells were generated with the Edit-R Cas9 nuclease (Horizon Discovery) and crRNAs targeting LIMD1 exon 1. crRNAs had the following target sequences in LIMD1 exon 1: 5′-ATG​GAT​AAG​TAT​GAC​GAC​CT-3′, 5′-CTT​CCA​AGA​TCA​AAC​TC-3′, and 5′-CCC​GAG​TTT​GAG​GAA​ACT​CGC-3′.

### siRNA transfection

siRNAs were purchased from Horizon Discovery (On-TARGETplus SMARTpool) and Merck (Mission predesigned siRNA), and the siRNA panel for PD-L1 regulators was purchased from QIAGEN (see Table S1 for more details). Cells were transfected using DharmaFECT1 transfection reagent (Horizon Discovery) or Lipofectamine RNAiMax (Life Technologies) according to the manufacturer’s instructions.

Table S1. siRNAs and primary antibodies used.

### PD-L1 3′UTR luciferase reporter assay

psiCheck2 reporter for PD-L1 3’UTR is described elsewhere (Yee et al, 2017). Cells were transfected with siRNAs at 30 nM final concentration and 48 h later transfected with psiCheck2 empty vector (VO) or PD-L1 3′ UTR. 24 h post-transfection, cells were lysed in 1X passive lysis buffer (Promega) and 10 μl of lysate was transferred to white 96-well microplates for dual-luciferase assay (Promega), according to the manufacturer’s instructions. Luciferase activities were measured on the BMG FLUOstar Omega plate reader.

### PD-L1 promoter luciferase assay

pNL-PD-L1 promoter reporter was created by cloning the −1,340 to +171 region of the *CD274* gene into the pNanoLuc vector (Promega) upstream of the NanoLuc cassette using *KpnI* and *BglII* restriction enzymes. The promoter region was PCR-amplified from human dermal fibroblast genomic DNA with specific primers and Phusion Hot Start 2X Master Mix (New England Biolabs). After Sanger sequencing to confirm correct clones, plasmid DNA was midiprepped. For reporter gene assays, H1299 cells were reverse-transfected in 96-well plates with 25 nM Scr or LIMD1 SMARTpool siRNA using DharmaFECT1. After 72 h, cells were transfected with pNL empty vector or pNL-PD-L1 promoter reporters and pGL3 FFLuc for normalisation. Six hours post-transfection, cells were treated with 1 ng/ml IFNγ or vehicle overnight. The next day, cells were harvested in 1X passive lysis buffer and lysates were assayed for Nano luciferase and firefly luciferase activities using Nano-Glo Dual-Luciferase Reporter Assay System. Luciferase activities were measured on the BMG FLUOstar Omega plate reader.

### Western blotting

Samples were prepared using RIPA buffer (150 mM NaCl, 50 mM Tris–HCl, pH 8, 1 mM EDTA, 0.1% SDS, 1% NP-40, 0.5% sodium deoxycholate) containing protease and phosphatase inhibitors. Protein concentrations were determined with BCA (Pierce), and lysates were diluted in SDS Laemmli sample buffer with β-mercaptoethanol before boiling for 5 min. After SDS–PAGE, proteins were transferred onto PVDF membranes (Merck) and blocked using 5% non-fat dried milk in PBS/Tween-20. Information on antibodies used is given in Table S1. After overnight incubation in the primary antibody, membranes were washed with PBS + 0.05% Tween-20 and incubated in HRP-conjugated secondary antibody before detection using Immobilon ECL substrate (Millipore) and imaged on Amersham ImageQuant 600 (GE Healthcare). Densitometry was performed with ImageJ software on both the protein of interest bands and corresponding bands for loading control proteins.

### 6His-tagged protein purification

Q5 site–directed mutagenesis (New England Biolabs) was used to change the HA-tag in pCMV-HA-PD-L1 to a 6His-tag. ARIH1 was cloned by PCR-amplifying the ARIH1 coding sequence from SAEC cDNA and inserting it into pcDNA3 using *EcoRI* and *NotI* restriction enzyme sites. The 6His-tag was added to the N terminus of ARIH1 by site-directed mutagenesis. pcDNA4-Xpr-6His-LIMD1 has been described previously (Foxler et al, 2012).

6His-tagged proteins were expressed in HEK293FT cells transfected with PEI MAX (Polysciences). Two days post-transfection, cells were collected by centrifugation and pellets were snap-frozen and stored at −80°C until required. Cell pellets were lysed in NPI-10 buffer (300 mM NaCl, 50 mM NaH_2_PO_4_, 10 mM imidazole, pH 8.0, 1% NP-40 containing protease inhibitor cocktail [Roche]). On ice, lysates were treated with Benzonase (Millipore) for 15 min and then cleared by centrifugation at 12,000*g* for 5 min at 4°C. After taking small volumes as input samples, cleared lysates were incubated overnight at 4°C with HIS-Select Nickel Magnetic Agarose Beads (Merck) previously equilibrated with NPI-10 buffer. The next day, beads were washed four times in NPI-20 buffer (300 mM NaCl, 50 mM NaH_2_PO_4_, 20 mM imidazole, pH 8.0) with 2-min rotation for each wash. 6His-tagged proteins were eluted from the beads by incubating for 5 min in NPI-500 buffer (300 mM NaCl, 50 mM NaH_2_PO_4_, 500 mM imidazole, pH 8.0). Buffer exchange was carried out to replace NPI-500 with 20 mM Tris, pH 7.5, 150 mM NaCl, 10% glycerol, 1 mM DTT by the use of Pierce Protein Concentrators with 10 kD MWCO. Elutions and input samples were subjected to Western blotting and Coomassie gel staining to confirm isolation of the desired proteins. Purified proteins were stored at −80°C until use.

### Real-time quantitative reverse transcription PCR

Total RNA was extracted from cells using TRI reagent (Sigma-Aldrich) according to the manufacturer’s instructions. After determining concentration with a NanoDrop spectrophotometer, 1 μg of RNA was treated with amplification-grade DNase I (Sigma-Aldrich) and diluted in nuclease-free water. Serial dilutions for RNA standard curves were prepared, and these along with samples and no template controls were used with GoTaq 1-Step qRT-PCR System (Promega) to assay PD-L1 and beta-actin in duplicate reactions (PD-L1 forward primer: 5′-TGGCATTTGCTGAACGCATTT-3′, PD-L1 reverse primer: 5′-AGTGCAGCCAGGTCTAATTGT-3′; beta-actin forward primer: 5′-CTGGAACGGTGAAGGTGACA-3′, beta-actin reverse primer: 5′-AAGGGACTTCCTGTAACAATGCA-3′). qRT-PCR was performed on Applied Biosystems QuantStudio 5 Real-Time PCR Machine, and data were analysed using Design and Analysis software (Applied Biosystems).

### Lentiviral transduction

A549, H1299, and RCC48 lentiviral lines were produced according to methods previously published (Naldini et al, 1996; Foxler et al, 2012). The murine LIMD1 open reading frames (ORFs) were PCR-amplified from splenocyte-derived cDNA. Amplification of ORFs used the following primer sequences: 5′-ATG​GAT​AAA​TAC​GAT​GAT​CTG​GGC-3′ (forward) and 5′-CTA​GAA​GTG​GTG​CTG​GTG​GA-3′ (reverse). The LIMD1 ORF including 3′ stop codon was cloned into the pCDH-EF1-FHC lentiviral expression vector by restriction digest and ligation with T4 DNA ligase (Thermo Fisher Scientific) to produce a pCDH-LIMD1 untagged expression vector. pCDH-EF1-FHC was a gift from Richard Wood at MD Anderson Cancer Center, University of Texas (plasmid #64874; Addgene).

### Immunoprecipitation

For immunoprecipitation of HA-PD-L1 (pCMV-HA-PD-L1, a gift from Mien-Chie Hung, plasmid #121492; Addgene) from A549 CRISPR-edited cells, plasmid DNA transfection was performed with Attractene Reagent (QIAGEN). 24 h post-transfection, cells were lysed in NP-40 lysis buffer with protease and phosphatase inhibitors, and lysates were cleared by centrifugation at 14,000*g* for 5 min at 4°C. Input samples were prepared and incubated with antibody overnight at 4°C. The next day, Protein G Mag Sepharose beads (Cytiva) were equilibrated in NP-40 buffer before adding to lysates. Bead/sample mixtures were incubated at room temperature for 1–2 h with rotation, before washing with NP-40 buffer (x4). Proteins were eluted from beads by incubation for 5 min with 0.1 M glycine, pH 2.5. Elutions were neutralised with 1 M Tris–HCl, pH 8, diluted, and boiled with SDS sample buffer. Input and elution samples were subjected to SDS–PAGE and transferred to PVDF membranes for immunoblotting.

### Proximity ligation assay

Cells were cultured on chamber slides (Falcon) coated with 0.1% gelatine. During seeding, cells were reverse-transfected with siRNA using Lipofectamine RNAiMax (Life Technologies). Three days post-transfection, cells were treated with 0.5 ng/ml IFNγ for 24 h before fixation in 4% formaldehyde for 10 min. Formaldehyde was removed with a PBS wash before permeabilising cells with 0.2% Triton X-100/PBS for 2 min. Cells were then incubated in a blocking solution (NaveniFlex Cell MR Kit, Navinci Diagnostics) for 1 h at 37°C in a humidified chamber. Primary antibodies diluted in blocking solution were then incubated with cells overnight at 37°C in a humidified chamber. Where no primary antibodies were included, only blocking solution was used. IgG isotype antibody was used as a negative control. The subsequent steps were performed according to the manufacturer’s instructions for the NaveniFlex Cell MR kit, with additional stains for the actin cytoskeleton using phalloidin-AF488 (Cell Signaling Technologies) and DAPI (Merck) for nuclei. After salt removal with distilled H_2_O, coverslips were mounted using ProLong Glass Antifade Mountant (Thermo Fisher). Slides were imaged on a Zeiss LSM 880 confocal microscope with a Plan-Apochromat 40×/1.3NA oil immersion objective lens. Excitation/emission wavelengths are as follows: DAPI: 405/444 nm; phalloidin: 488/512 nm; PLA signal: 561/606 nm. Images were processed using ZEN software to generate .tiff files. PLA focus counts per cell were acquired from the .tiff images using CellProfiler software (version 4.2.4). Nuclei were identified as PrimaryObjects from DAPI staining. Cells were identified by the actin staining from nuclei as input objects with the watershed–gradient method and a global thresholding strategy. PLA foci were identified as having typical diameters between 3 and 15 pixel units. Global thresholding with minimum cross-entropy was used for PLA foci. Parent objects (cells) were related to child objects (PLA foci) using the RelateObjects module to give PLA focus counts per cell. These were then plotted using GraphPad Prism software with the appropriate statistical test performed on the data.

### PD-L1 ubiquitination assay

Cells grown in 10-cm dishes were treated with 2 ng/ml IFNγ overnight and then for 6 h with 10 μM MG132. Cells were lysed in RIPA buffer containing 10 mM N-ethylmaleimide. Lysates were cleared by centrifugation at 14,000*g* for 5 min at 4°C, and then, supernatants were transferred to new tubes. Input samples were prepared, and then, lysates were incubated overnight at 4^o^C with anti-PD-L1 antibody (ab213524; Abcam) or rabbit IgG isotype control (Cell Signaling Technologies). The next day, Protein A Dynabeads (Life Technologies) were equilibrated in lysis buffer and added to lysates to capture antibody–antigen complexes for 1 h at room temperature with gentle agitation. Beads were then washed with RIPA buffer once, four times with high-salt RIPA buffer (600 mM NaCl), and once more with RIPA buffer. Proteins were eluted from beads by boiling with 1X SDS sample buffer. Input and elution samples were subjected to SDS–PAGE and transferred to PVDF membranes for immunoblotting for ubiquitin and PD-L1.

### GST-LIMD1 expression, purification, and direct binding assay

pGEX6P1-LIMD1 has been described previously (Sharp et al, 2004). This construct, as well as empty vector, was used to transform BL21 DE3 *Escherichia coli* (New England Biolabs), and transformants were selected on LB agar plates containing 50 μg/ml ampicillin. Colonies were picked into 5-ml LB ampicillin starter culture for overnight incubation at 37°C. The next day, 3 ml of starter culture was used to inoculate 200 ml of LB ampicillin and the culture was incubated at 37°C until OD600 reached ∼0.6. The cultures were then cooled on ice for 10 min before adding IPTG to a final concentration of 0.5 mM and incubating the culture overnight at 20°C to induce protein expression. Bacteria were pelleted by centrifugation for 15 min at 3,500*g*, and pellets were stored at −80°C until use.

For lysis, pellets were weighed and 2.8× mass of NP-40 lysis buffer was used to resuspend (e.g., for a 300 mg pellet, 840 μl of lysis buffer was added). Bacteria were sonicated at 4°C at 25% amplitude for 10-s ON and 30-s OFF for a total ON time of 90s. Lysates were then centrifuged at 20,000*g* for 10 min at 4°C. Supernatants were aliquoted and stored at −80°C, and pellets were discarded.

GST proteins were captured by incubating bacterial lysates with pre-equilibrated glutathione Sepharose beads (GE Healthcare) in binding buffer (PBS + 0.1% Triton X-100 + 5% glycerol [vol/vol]) for 45 min at 4°C. Beads were then washed three times in binding buffer. To the captured GST proteins on beads, and a beads-only control, purified 6His-ARIH1 was added. Input samples were taken at this point, and the mixtures were incubated with rotation overnight at 4°C. The next day, beads were washed four times in binding buffer and proteins were eluted from beads in a buffer containing 50 mM reduced glutathione. Elutions were mixed with 5X SDS sample buffer and subjected to SDS–PAGE alongside input samples.

### In vitro ubiquitination assay

In vitro ubiquitination assays were set up using Ubiquitin Activating Kit (BML-U0400A; Enzo Biochem, Inc.). Reactions were set up according to the kit instructions and incubated for 4 h at 37°C. Reactions were quenched by adding an equal volume of 2X non-reducing sample buffer included in the kit. Quenched reactions were stored at −20°C until subjected to SDS–PAGE and Western blotting.

### Flow cytometry

For analysis of surface PD-L1 expression in adherent cancer cell lines, wells were washed once with PBS before detaching cells with Accutase (STEMCELL Technologies). Single-cell suspensions were washed in PBS/1% FCS before staining with APC-conjugated anti-human-PD-L1 (29E.2A3; BioLegend), anti-mouse-PD-L1 (10F.9G2; BioLegend), or respective IgG controls. For analysis of T-cell CD25 expression, suspension cells were collected, washed, and stained with APC-conjugated anti-CD8 (SK1; BioLegend), PerCP-conjugated anti-CD4 (OKT4; BioLegend), and BV605-conjugated anti-CD25 (BC96; BioLegend). Cells were stained for 30 min on ice and washed in PBS before analysis using FACSAria II (BD). FCS files were analysed using FlowJo V10.7 (TreeStar).

### PBMC co-culture

96-well plates were coated with Ultra-LEAF anti-CD3 (OKT3; BioLegend) diluted in PBS. Plates were sealed and incubated at 37°C for 2 h. PBMCs were prepared from leukocyte cones from healthy blood donors under the East London and The City ethics approval licence 05/Q0605/140, awarded by the local research ethics committee. PBMCs were harvested, centrifuged, and resuspended in RPMI-1640/10% FCS growth media supplemented with recombinant human interleukin-2 (10 ng/ml, Roche) before incubation at 37°C, 5% CO_2_ for 3 d. 3 × 10^3^ target A549 cells were seeded into each well and incubated for 5 h or until adhered. Anti-CD3–activated PBMCs were harvested from 96-well plates and resuspended to 15 × 10^4^ cells/ml in complete media. A549-H2B-mCherry target cells expressing a nuclear fluorescent reporter protein were seeded in growth media containing hIL-2 (final concentration 10 ng/ml). For studies of immune checkpoint function, cultures were treated with pembrolizumab (anti-PD-1, Merck), atezolizumab (anti-PD-L1, Genentech), or humanised IgG1 control (InVivoMAb, BioXcell) at 10 μg/ml. 50 × 10^3^ PBMCs were then seeded into cultures at predetermined target-to-effector ratios, to a final 200 μl culture volume. Images were captured using the IncuCyte ZOOM live-cell imaging system (Essen Biosciences).

### 3D culture/spheroid experiments

For 3D spheroid assays, 500 viable target A549-mKate cells expressing a cytosolic reporter protein were seeded into ultra-low attachment plates (Corning) and cultured for 7 d until spheroids had formed. Cell culture media were removed carefully, and 1 × 10^5^ activated PBMCs were seeded into wells containing spheroids. Images were captured using the IncuCyte S3 live-cell imaging system (Essen Biosciences). For measurement of cell death within co-cultures, media were supplemented with CellTox Green reagent (Promega), with red and green fluorescence measured at 650 and 530 nm, respectively.

### TRACERx data analysis

TRACERx gene expression and clinicopathological data were obtained from Martinez-Ruiz et al (2023). LIMD1 copy-number information was obtained from Frankell et al (2023). Tumours were considered to have “Clonal” loss if there was evidence of copy-number loss relative to ploidy throughout the LIMD1 locus in all constituent regions of a tumour. Tumours were considered as having “Subclonal” loss if there was evidence of copy-number loss relative to ploidy anywhere in the LIMD1 locus in at least one constituent region of a tumour, as well as evidence of a non-loss copy-number state relative to ploidy anywhere in the LIMD1 locus in at least one constituent region of a tumour. Tumours were considered as having “No loss” if there was no evidence of copy-number loss relative to ploidy at any point of the LIMD1 locus in any of the constituent regions of a tumour. Gene expression data were VST-transformed for downstream analysis of the impact of LIMD1 gene expression. Where indicated, Wilcoxon tests performed in this work are two-sided, using the function wilcox.test () in base R. Timing of single-nucleotide variants and copy-number alterations (amplifications and LOH events) in LUAD was obtained with a Bradley–Terry model applied to clone-specific copy numbers inferred by the allele-specific phylogenetic analysis of copy-number alterations algorithm from TRACERx-421 data (Pawlik et al, 2025).

### CPI data analysis

Three cohorts were used for the ICB analysis—26 patients from the Samsung Medical Centre (Jung et al, 2019), 323 patients from the OAK clinical trial (Patil et al, 2022), and 57 from the POPLAR trial (Banchereau et al, 2021). All patients are treated with anti-PD-1 or anti-PD-L1 monotherapy. Clinical data such as treatment type were collected, and response outcomes were harmonised using the RECIST criteria (Eisenhauer et al, 2009). RNA-sequencing data were derived from the studies in a FASTQ format and then processed through the RIMA pipeline (Yang et al, 2023). Gene quantification was conducted using tximport (Salmon) and was TPM-normalised for downstream analysis.

### Human participation ethics declarations

#### The TRACERx 421 cohort

The TRACERx study (https://clinicaltrials.gov/ct2/show/NCT01888601) is a prospective observational cohort study that aims to transform our understanding of NSCLC, the design of which has been approved by the NHS Health Research Authority: Camden & Kings Cross Research Ethics Committee (13/LO/1546). Informed consent for entry into the TRACERx study was mandatory and obtained from every patient. All patients were assigned a study identity number that was known to the patient. These were subsequently converted to linked study identifiers (containing the CRUK prefix) such that patients could not identify themselves in study publications. All human samples (tissue and blood) were linked to the study identity number and barcoded such that they were anonymised and tracked on a centralised database, which was overseen by the study sponsor (UCL Clinical Trials Centre) only. The cohort represents the first 421 patients whose samples were received for processing and who met the eligibility criteria.

#### CPI data cohorts

For data obtained from Jung et al (2019), informed consent was obtained. This study was approved by the institutional review board at Samsung Medical Center (2018-03-130 and 2013-10-112).

For data obtained from Patil et al (2022) and Banchereau et al (2021), both the OAK and POPLAR studies were performed in full accordance with the guidelines for Good Clinical Practice and the Declaration of Helsinki, and all patients gave written informed consent. Protocol approval was obtained from independent ethics committees for each participating site for both studies, and an independent data monitoring committee reviewed the safety data.

## Supplementary Material

Reviewer comments

## Data Availability

All data supporting the findings of this study are available within the article and its Supplementary Information. Any additional information required is available from the lead contact upon request. No new code was generated in this article.
