# Supplementary material for: Clonal LIMD1 loss drives PD-L1 immune evasion via ARIH1-dependent ubiquitination in lung cancer
Source: Life Sci Alliance. 2026 Aug 7;9(10):e202603812. doi: 10.26508/lsa.202603812 (PMC13451150; doi:10.26508/lsa.202603812)

Source Data Figure 3

Panel A

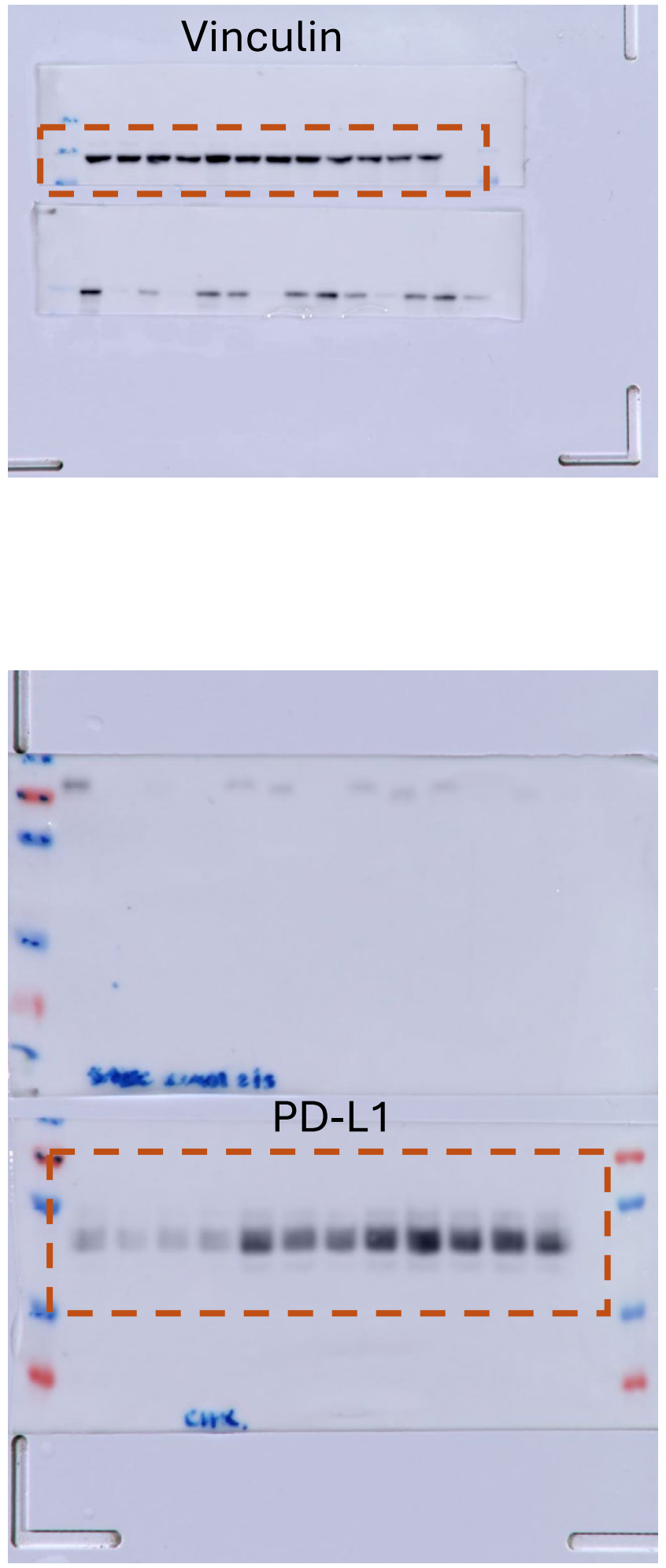

Panel B

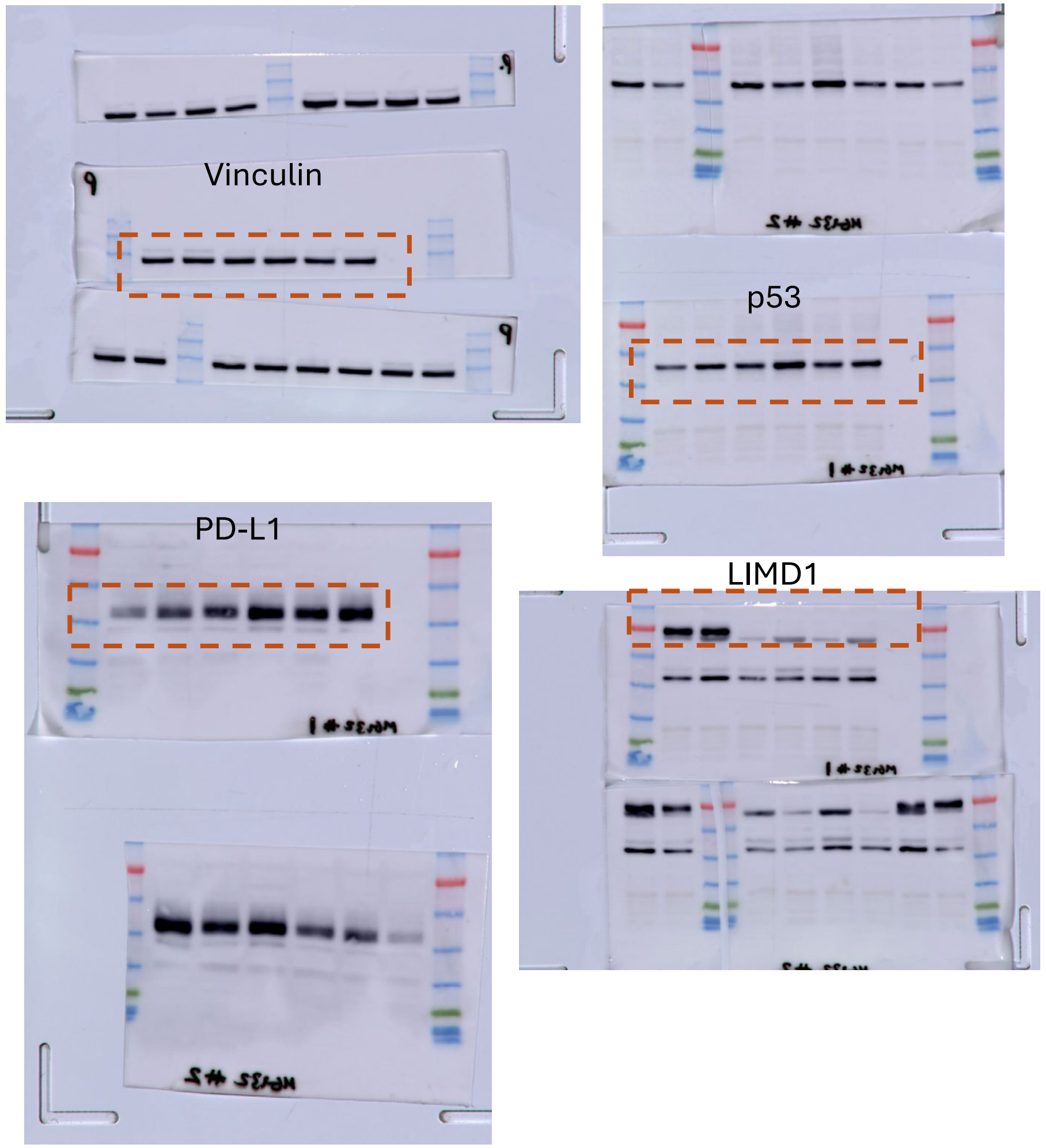

Panel C

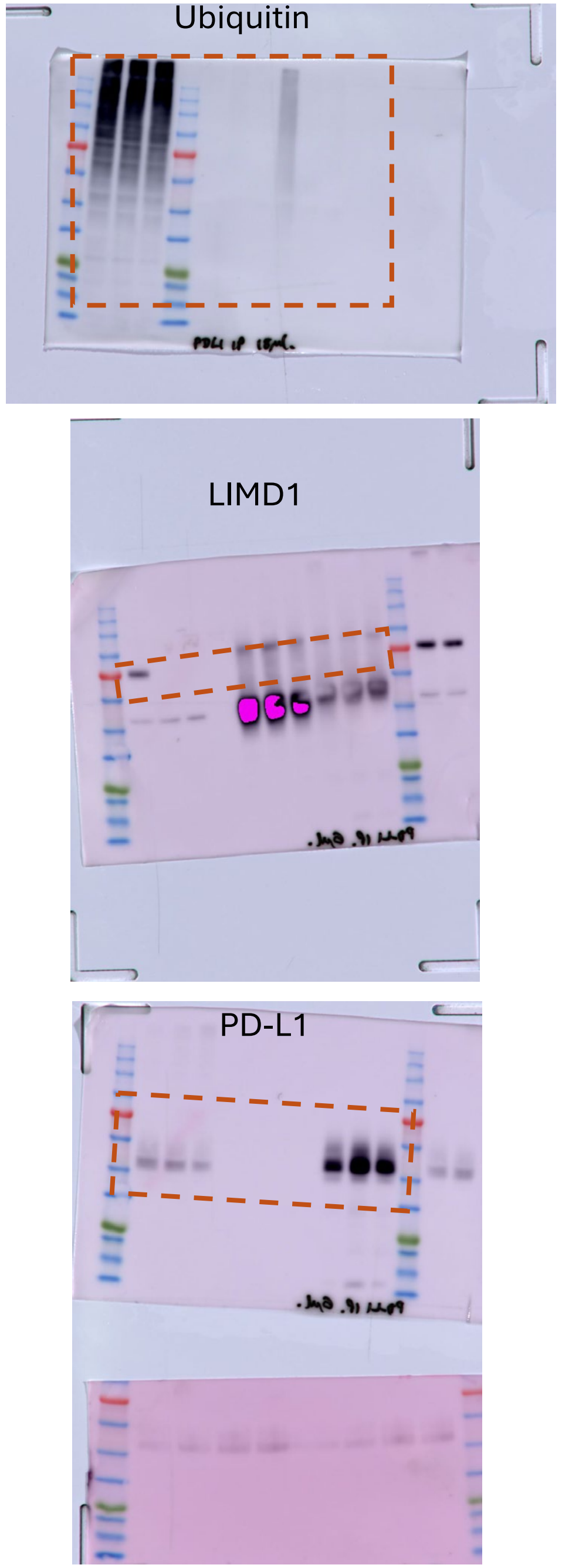

Panel E

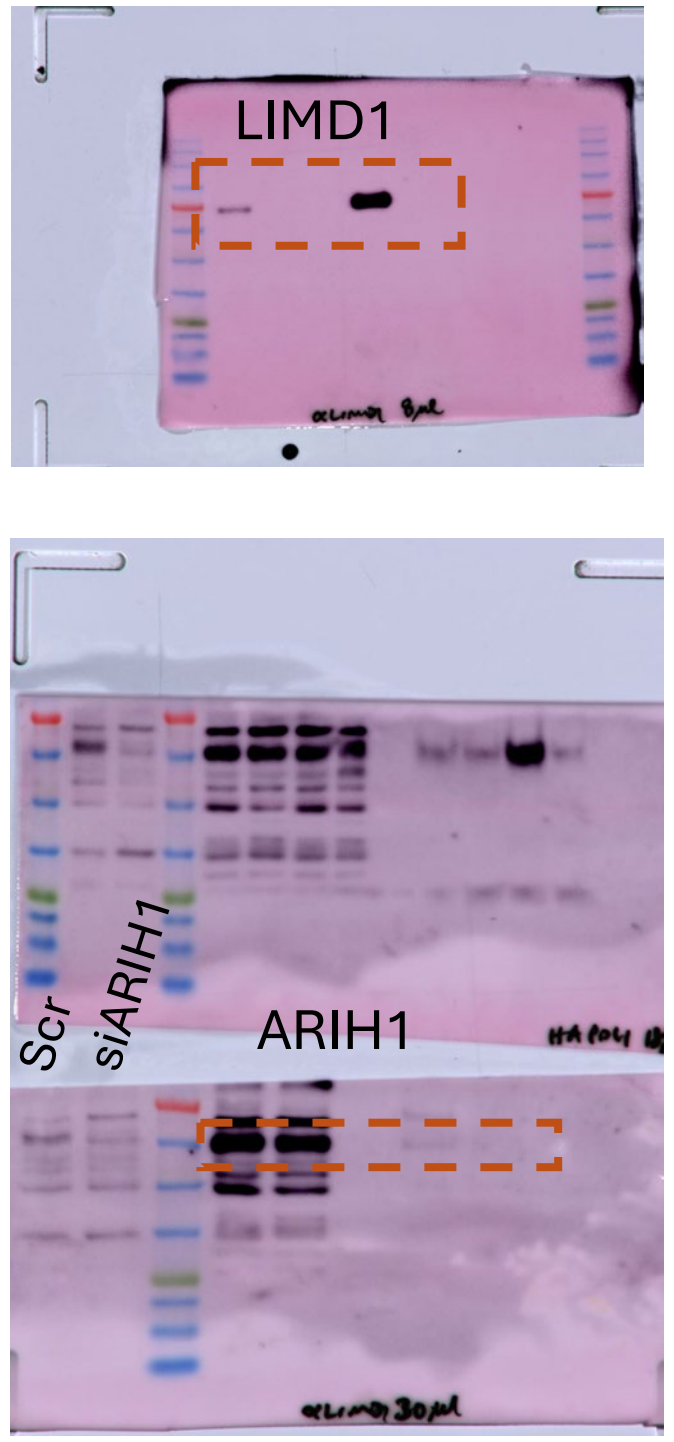

Source Data Figure 3

Panel F

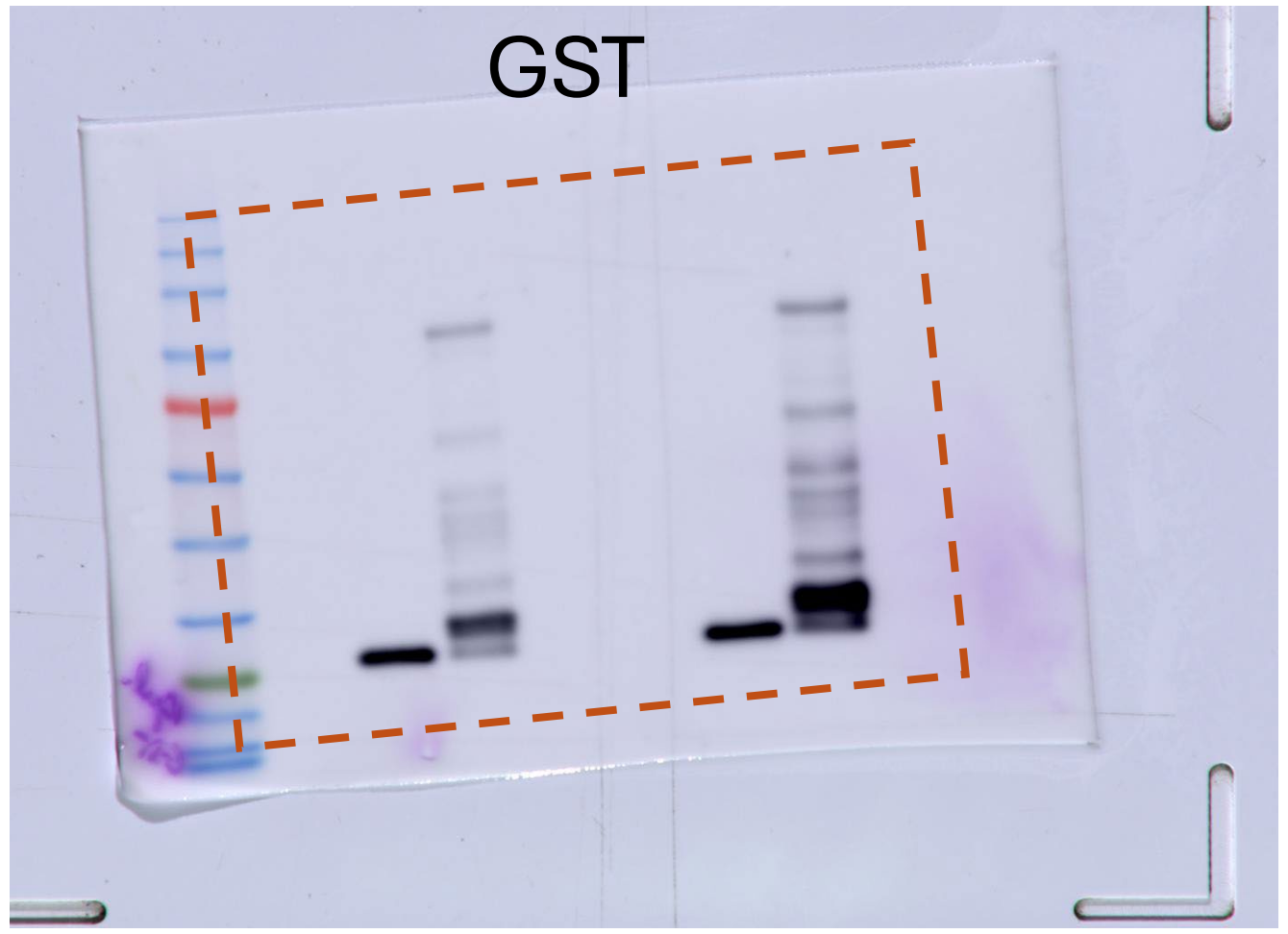

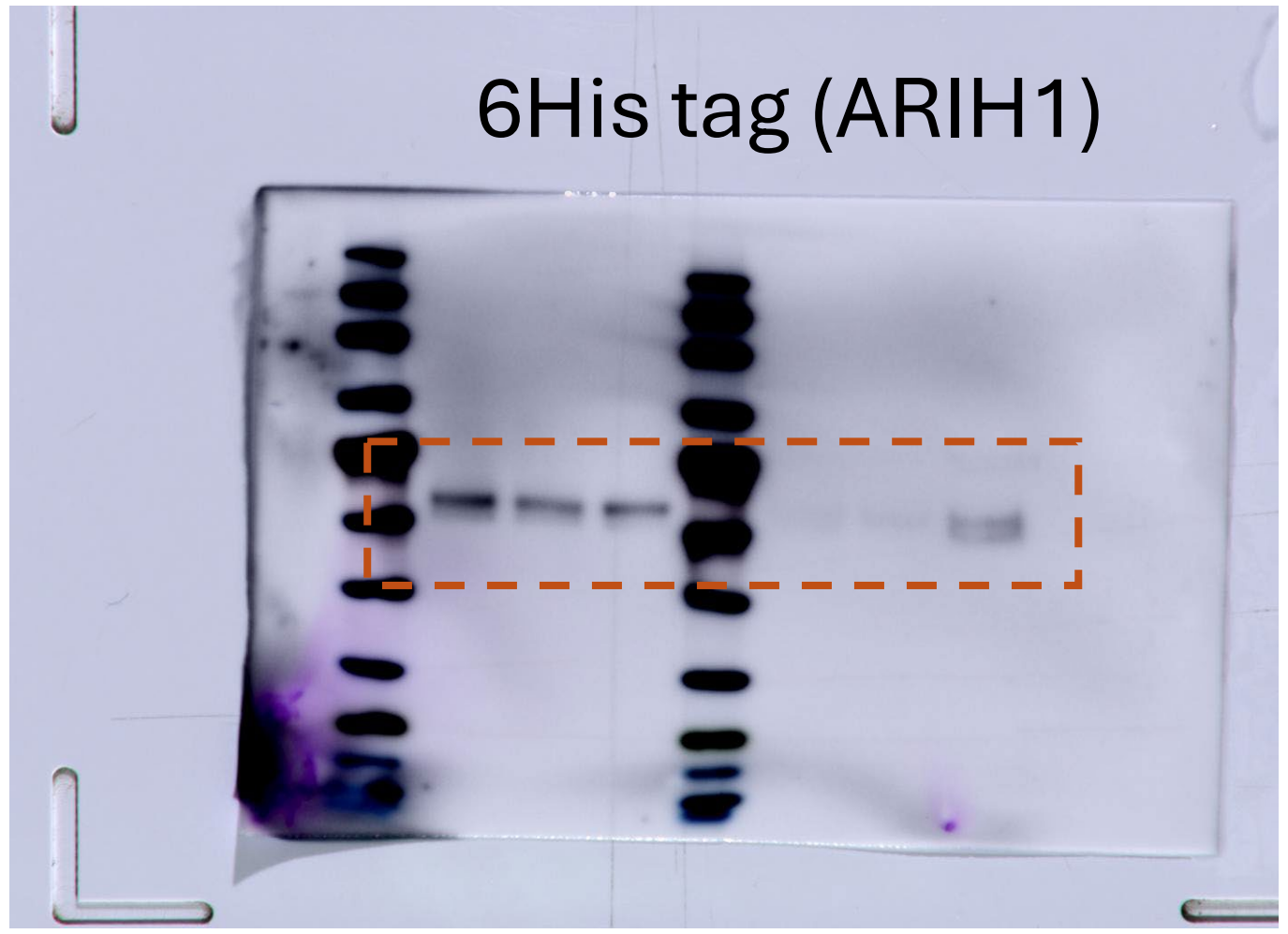

Panel G

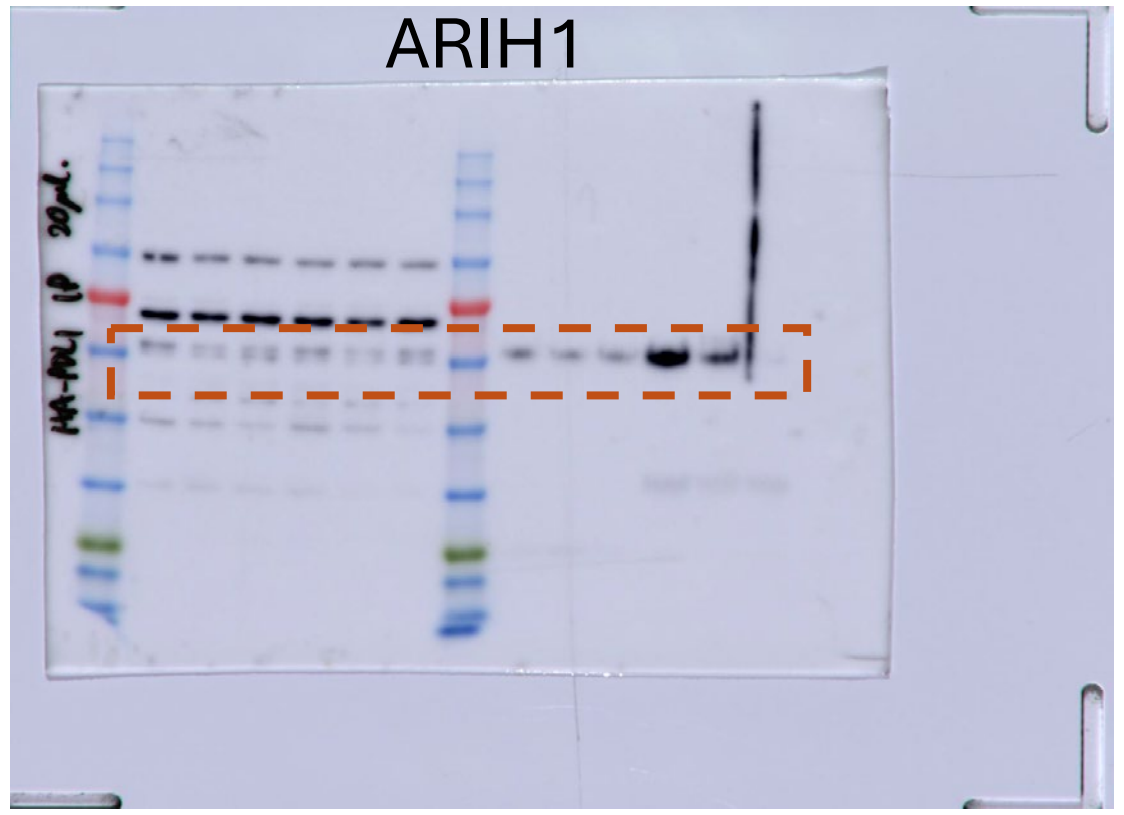

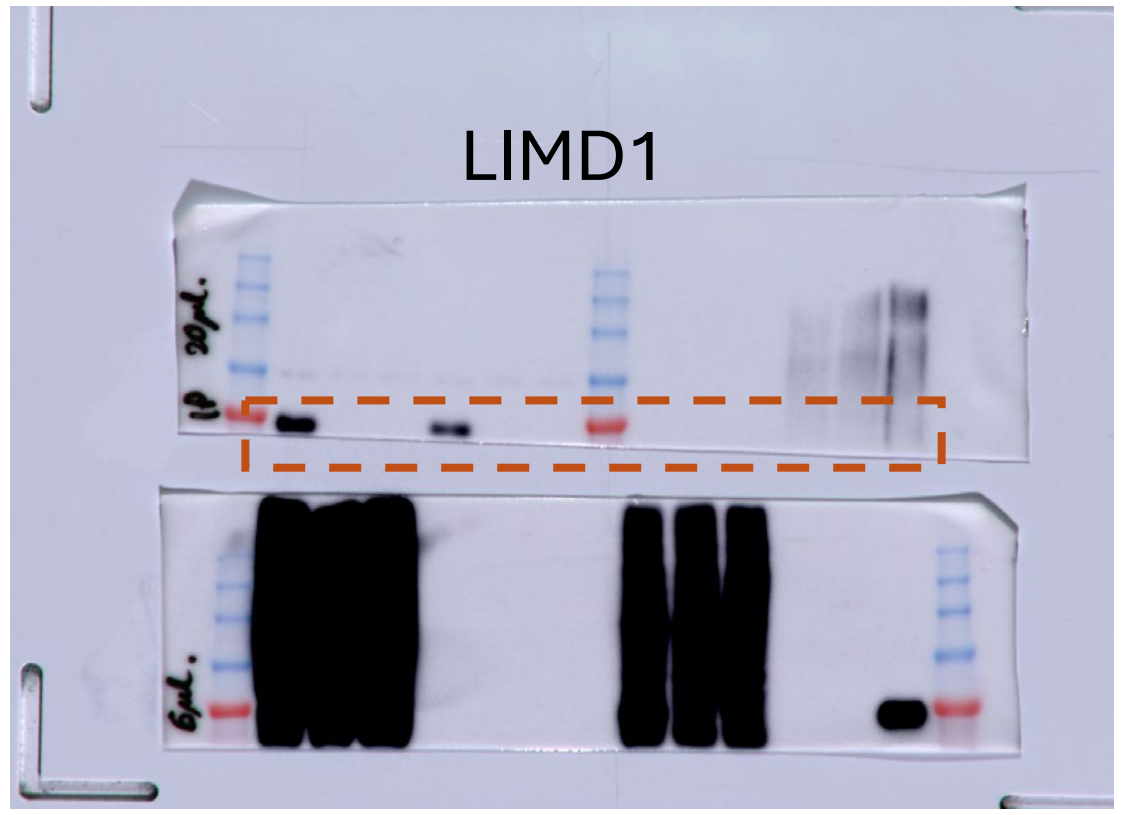

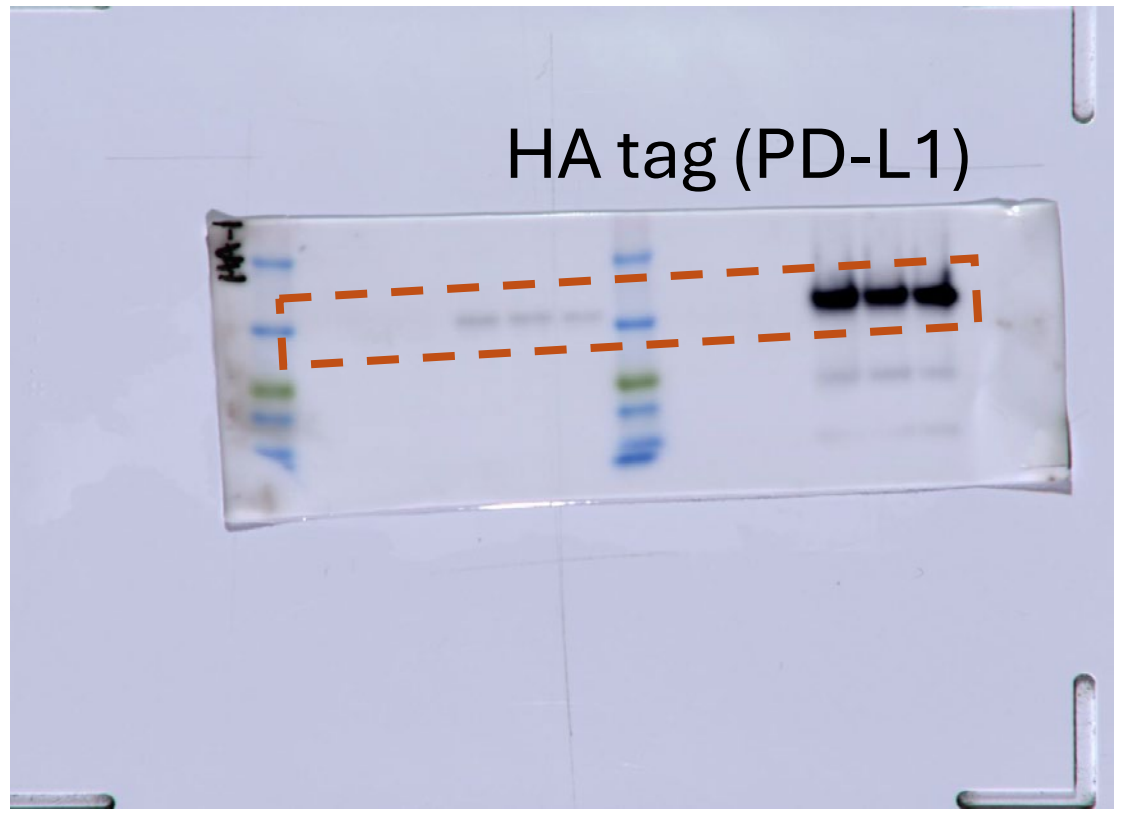

Panel I

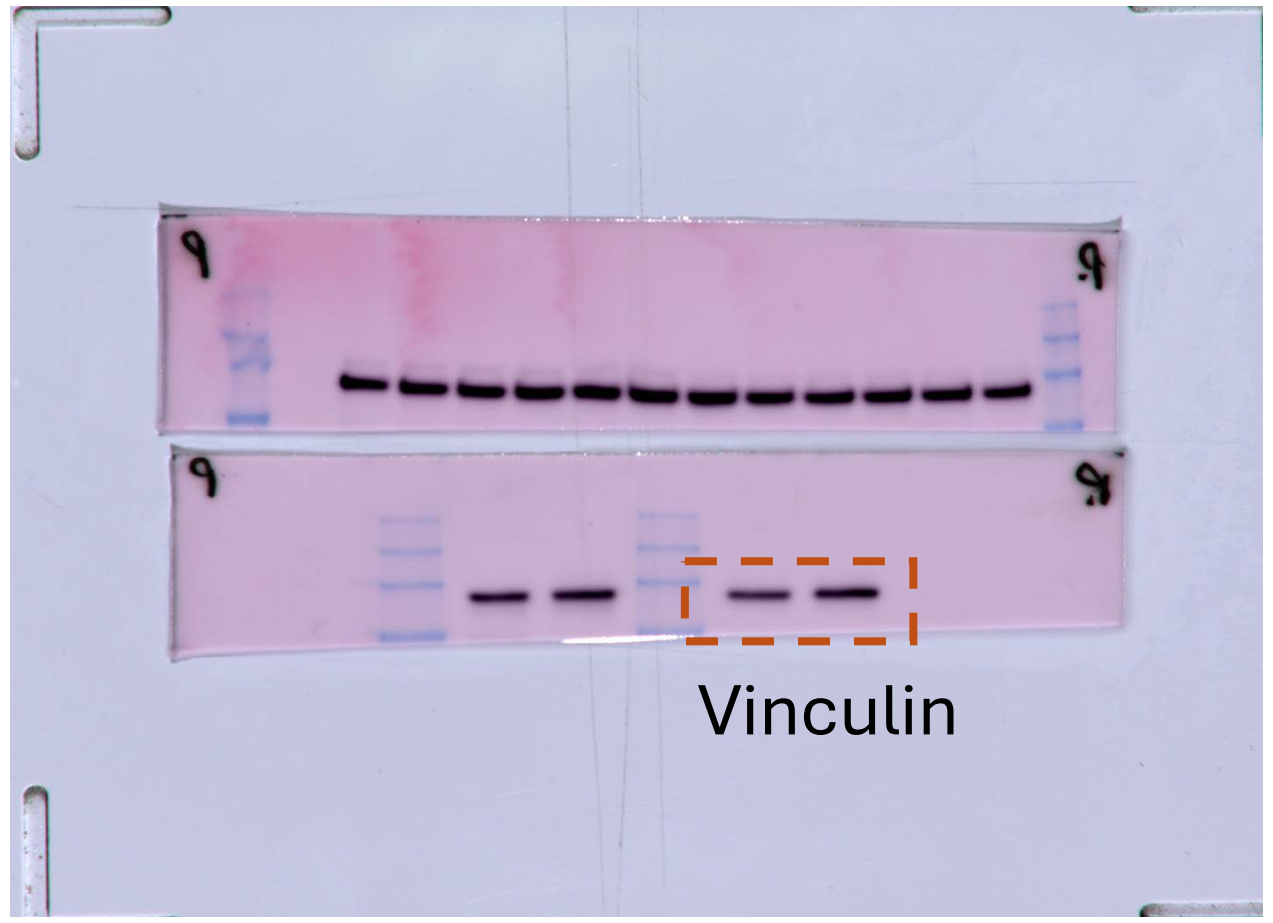

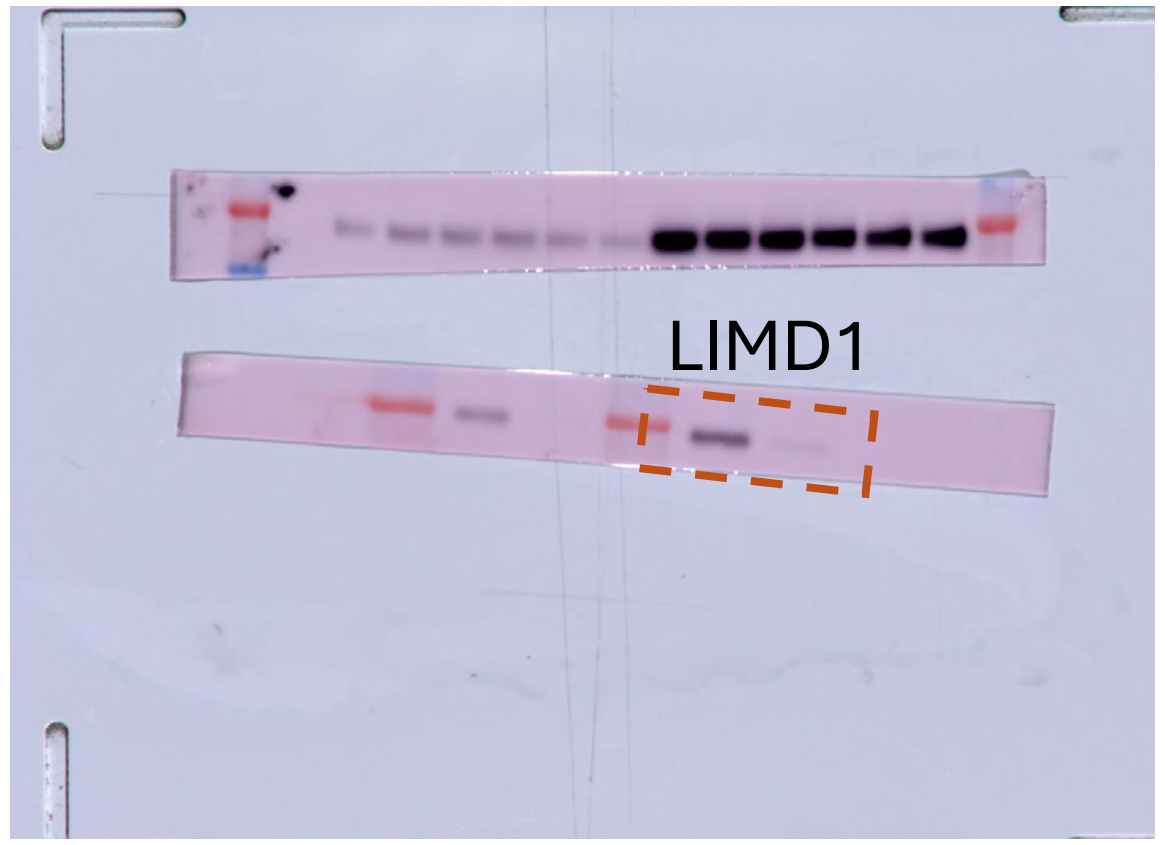

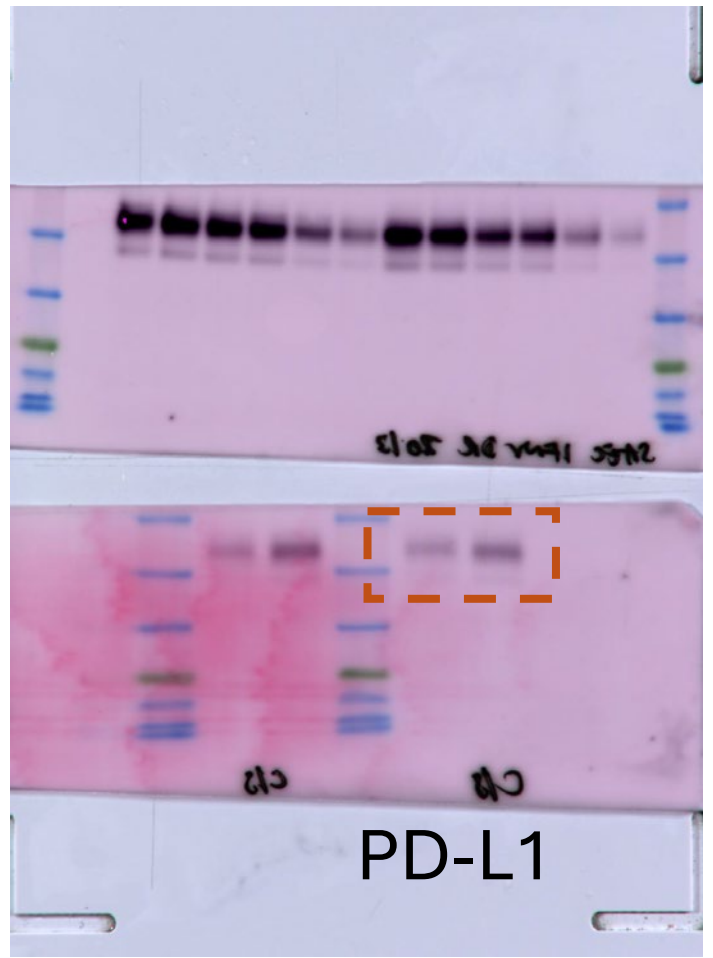

Supplement: Supplementary file 3 [file LSA-2026-03812_SdataF3.pdf]
