# Supplementary material for: Clonal LIMD1 loss drives PD-L1 immune evasion via ARIH1-dependent ubiquitination in lung cancer
Source: Life Sci Alliance. 2026 Aug 7;9(10):e202603812. doi: 10.26508/lsa.202603812 (PMC13451150; doi:10.26508/lsa.202603812)
Supplement: Supplementary file 4 [file LSA-2026-03812_review_history.pdf]

Clonal LIMD1 loss drives PD-L1 immune evasion via ARIH1-dependent ubiquitination in lung cancer

Kunal Shah, Paul Kennedy, James Black, Piotr Pawlik, Kevin Litchfield, Krupa Thakkar, Maria Contreras G., Kirstin Brooksbank, Oliver Yuan, Paul Grevitt, Sarah Charrot, Jeff Davies, Lekh N. Dahal, Dimitris Lagos, Nicholas McGranahan, and Tyson Sharp
DOI: https://doi.org/10.26508/lsa.202603812

Corresponding author(s): Tyson Sharp, Queen Mary University of London and Kunal Shah, Queen Mary University of London

Review Timeline:

Submission Date:	2026-06-11
Editorial Decision:	2026-07-10
Revision Received:	2026-07-23
Accepted:	2026-07-28

Scientific Editor: Sarita Hebbar

Transaction Report:

(Note: With the exception of the correction of typographical or spelling errors that could be a source of ambiguity, letters and reports are not edited. The original formatting of letters and referee reports may not be reflected in this compilation.)

Please note that the manuscript was previously reviewed at another journal and the reports were taken into account in the decision-making process at *Life Science Alliance*. Since the original reviews are not subject to Life Science Alliance's transparent review process policy, the reports and author response cannot be published.

July 10, 2026

RE: Life Science Alliance Manuscript #LSA-2026-03812-T

Prof. Tyson Valentine Sharp
t.sharp@qmul.ac.uk
Centre for Molecular Oncology
Barts Cancer Institute
Queen Mary, University of London
London, Not applicable EC1M 6BQ
UNITED KINGDOM

Dear Dr. Sharp,

Thank you for submitting your revised manuscript entitled "Clonal LIMD1 loss drives PD-L1 immune evasion via ARIH1-dependent ubiquitination in lung cancer".

Your manuscript, the submitted reviews, and point-by-point rebuttal were assessed by LSA's editorial team and an expert who acted as an arbitrator. This expert expressed interest in these findings and has ruled out any further additional in-vivo/mouse experiments.
In line with this advice, we we would be happy to publish your paper in Life Science Alliance pending resolution of the points immediately below, and final revisions necessary to meet our formatting guidelines.

We agree with the comments in the previous review (submitted, and explained on 29.6.2026) that your ms must address the following with minor edits:

-Wherever possible, refrain from references to "context-dependent" regulation of PD-L1 by LIMD1 as translational and post-translational regulation were not ascribed to different contexts. The results and/or discussion should comment on why different cell lines would exhibit different modes of regulation.
-In the results section, the discrepancy in CD25 levels noted by the reviewer should be commented on with the proposed rationale that this was due to different PBMC donors.
-The possibility that LIMD1 loss alters basal rates of cell proliferation and/or cell death was not tested in this work. This must be mentioned in the discussion or prior evidence on this point should be referenced. The change in cell death in Figure 4H, as commented on by the reviewer, must be explicitly discussed in the results for this section.
-In the absence of truncation mutants of LIMD1 testing binding to PD-L1 and ARIH1, rephrase its "scaffolding" function as conjecture awaiting formal confirmation.
-The western blots for A549 cells in Figures 1A and S1 A appear to be the same. Please verify and include the correct blots for both figures.

Please submit a revised manuscript with the changes highlighted. Please also include a point-by-point response letter commenting on the above points.

MANUSCRIPT ORGANIZATION AND FORMATTING:

To avoid unnecessary delays in the acceptance and publication of your paper, please read the following information carefully. Full guidelines are available on our Instructions for Authors page, https://www.life-science-alliance.org/authors

Submission of a paper that does not conform to Life Science Alliance guidelines will delay the acceptance of your manuscript.

-Please remove all sub-headings from the Discussion section and integrate the following sub-sections into one discussion section (conclusion, future directions and clinical/biological insight).
-Kindly include antibody concentration in Table S1 and kindly callout to the table in the methods section
-Please expand on details for imaging include NA for objectives and excitation/emission information.
-Please add molecular weight markers for Figure S1A.
-Please merge supplementary methods information with the main section on methods.
-Please provide sequence information for the targeting of exon 1 and related information for siRNA.
-Please elaborate on the source of PBMCs in the PBMC-co-culture experiments. Kindly provide the necessary declaration for harvesting these cells.
-Kindly elaborate on the purified proteins used for the in-vitro Ubiqiuitnation Assays or call out to the relevant section if already

provided.
-Please include callout for: Table S1, Figures 2I-J, 3J and S2C.
-Please remove the callout for Figure S2H (which does not exist).
-Please upload your main manuscript text as an editable doc file.
-Please upload all figure files as individual ones, including the supplementary figure files; all figure legends should only appear in the main manuscript file. Please remove figures from main manuscript file.
-Please add your main, supplementary figure, and table legends to the main manuscript text after the references section.
-please upload your Supporting Table in editable .doc or excel format.
-Please add a Category for your manuscript in our system.
-please add Keywords for your manuscript in our system.
-Please add a Summary Blurb/Alternate Abstract in our system.
-Please add the X and Bluesky handles of your host institute/organization, as well as your own, and/or one of the authors, in our system.
-please add ORCID ID for secondary corresponding author--you should have received instructions on how to do so.
-Please be sure that the authorship listing and order are correct and match between the system and manuscript file.
-Please consult our manuscript preparation guidelines https://www.life-science-alliance.org/manuscript-prep and make sure your manuscript sections are in the correct order
-Please remove ORCID IDs from manuscript cover page.
-Please add an Author Contributions section to your main manuscript text.
-Please use the [10 author names et al.] format in your references (i.e., limit the author names to the first 10).
-The "Data Availability" section should be placed after the Materials & Methods section. Please consult our guidelines at https://www.life-science-alliance.org/manuscript-prep#format
-Please be sure that the authorship listing and order is correct

We encourage our authors to provide original source data, particularly uncropped/-processed electrophoretic blots and spreadsheets for the main figures of the manuscript. If you would like to add source data, we would welcome one PDF/Excel-file per figure for this information. These files will be linked as supplementary "Source Data" files.

It is Life Science Alliance policy that if requested, original data images must be made available to the editors. Failure to provide original images upon request will result in unavoidable delays in publication. Please ensure that you have access to all original data images prior to final submission.

If you are planning a press release on your work, please inform us immediately to allow informing our production team and scheduling a release date.

We welcome submissions of potential cover images for the issue of LSA in which your work would appear. If you have high quality images associated with this work, please feel free to email these, with a caption, to the journal office.

LSA encourages authors to provide a 30-60 second video where the study is briefly explained. These videos will be appear embedded with the manuscript online at Life Science Alliance, and on social media to promote the published paper and authors (for examples, see https://docs.google.com/document/d/1-UWCfbE4pGcDdcgzcmiuJl2XMBJnxKYeqRvLLrLSo8s/edit?usp=sharing). Corresponding or first-authors are welcome to submit the video. Please submit only one video per manuscript. The video can be emailed to contact@life-science-alliance.org

FINAL FILES:

To upload the final version of your manuscript, please log in to your account: https://lsa.msubmit.net/cgi-bin/main.plex
You will be guided to complete the submission of your revised manuscript and to fill in all necessary information. Please get in touch in case you do not know or remember your login name.

The following items are required for acceptance.

-- An editable version of the final text (.DOC or .DOCX) is needed for copyediting (no PDFs).

-- High-resolution figure, supplementary figure and video files uploaded as individual files: See our detailed guidelines for preparing your production-ready images, https://www.life-science-alliance.org/authors

-- Summary blurb (enter in submission system): A short text summarizing in a single sentence the study (max. 200 characters including spaces). This text is used in conjunction with the titles of papers, hence should be informative and complementary to the title. It should describe the context and significance of the findings for a general readership; it should be written in the present tense and refer to the work in the third person. Author names should not be mentioned.

The license to publish form must be signed before your manuscript can be sent to production. A link to the license to publish form will be available to the corresponding author only. Please take a moment to check your funder requirements.

Reviews, decision letters, and point-by-point responses associated with peer-review at Life Science Alliance will be published, alongside the manuscript. If you do want to opt out of having the reviewer reports and your point-by-point responses displayed, please let us know immediately.

Thank you for your attention to these final processing requirements. Please revise and format the manuscript and upload materials as soon as you are able.

Thank you for this interesting contribution to the literature. We look forward to publishing your paper in Life Science Alliance.

Sincerely,

Sarita Hebbar, PhD
Scientific Editor
Life Science Alliance
http://www.lsajournal.org

REF *LSA-2026-03812-TR*

Dear Sarita,

We are pleased that our manuscript has been accepted for publication pending the changes you requested. Please see below our point-by-point responses to the minor edits you require:

Our response to the comments:

-Wherever possible, refrain from references to "context-dependent" regulation of PD-L1 by LIMD1 as translational and post-translational regulation were not ascribed to different contexts. The results and/or discussion should comment on why different cell lines would exhibit different modes of regulation.

We have removed the phrase "context-dependent" from the abstract and the other parts of the manuscript (page 3 lines 5-8, page 6 line 2-3, page 10 line 10, page 13 line 10) and on page 19 lines 2-5, we have added in the Discussion a comment on why different cell lines may exhibit different modes of regulation.

-In the results section, the discrepancy in CD25 levels noted by the reviewer should be commented on with the proposed rationale that this was due to different PBMC donors.

We have now mentioned on page 10 lines 22-23 the discrepancy in CD25 levels may be due to different PBMC donors. "*We note that the differing basal levels of CD25 on CD8+ T cells in figures 4D and 4E are likely due to different donors for PBMCs*."

-The possibility that LIMD1 loss alters basal rates of cell proliferation and/or cell death was not tested in this work. This must be mentioned in the discussion or prior evidence on this point should be referenced. The change in cell death in Figure 4H, as commented on by the reviewer, must be explicitly discussed in the results for this section.

We agree that in this study we did not examine if LIMD1 loss alters the basal rates of cell proliferation and/or cell death. However, this has been addressed in previous publications which we have now indicated (see below)

In the results section we have now included the following regarding Figure 4H (page 11 lines 7-13): "*We note that LIMD1$^{-/-}$ tumour cells showed a modestly increased death signal compared*

Queen Mary University of London

Barts Cancer Institute

Prof. Tyson Valentine Sharp
Centre Lead
Centre of Molecular and Cellular Biology
Barts Cancer Institute
Old Anatomy Building
Charterhouse Square
London EC1M 6BQ
bci.qmul.ac.uk
Email: t.sharp@qmul.ac.uk

with LIMD1$^{+/+}$ cells even in IgG-treated co-cultures. Because these cultures contained activated PBMCs, this condition does not measure spontaneous tumour-cell death in isolation. The increased death signal may therefore reflect basal immune-mediated killing and/or altered tumour-cell fitness associated with LIMD1 loss. Importantly, PD-L1 blockade further increased tumour-cell death preferentially in LIMD1$^{-/-}$ co-cultures, supporting the conclusion that elevated PD-L1 functionally restrains anti-tumour immune activity in LIMD1-deficient cells."

In the Discussion we have now referenced previously published work that demonstrate how LIMD1 affects cell proliferation and added the following (page 14 lines 4-13):

"A limitation of this study is that basal proliferation and spontaneous cell death of LIMD1+/+ and LIMD1−/− cells were not directly quantified under all co-culture conditions. Previous work demonstrated that LIMD1 interacts with pRB, represses E2F-dependent transcription and suppresses tumour growth, consistent with a role for LIMD1 in regulating cell-cycle progression and tumour-cell fitness. Therefore, intrinsic differences in proliferation or survival may contribute to the absolute tumour-cell death signals observed in co-culture. Nevertheless, because the increased death was measured in the presence of activated PBMCs and was further enhanced by PD-L1 blockade, the data remain consistent with a model in which LIMD1 loss promotes PD-L1-dependent immune suppression while leaving tumour cells susceptible to immune-mediated killing when this checkpoint is inhibited."

-In the absence of truncation mutants of LIMD1 testing binding to PD-L1 and ARIH1, rephrase its "scaffolding" function as conjecture awaiting formal confirmation.

We have now removed references to "scaffolding" when describing the interaction of LIMD1, ARIH1 and PD-L1.

-The western blots for A549 cells in Figures 1A and S1 A appear to be the same. Please verify and include the correct blots for both figures.

These western blots are different for different cells. In Figure 1A we have lentiviral lines for H1299 and RCC48 cells. In figure S1A, they are blots for A549 and H1299 lentiviral lines generated with independent shRNA sequences for LIMD1 (shLIMD1_2).

For the Manuscript Organization and Formatting, we have also addressed these points:

-Please remove all sub-headings from the Discussion section and integrate the following sub-sections into one discussion section (conclusion, future directions and clinical/biological insight). Done

Queen Mary University of London

Barts Cancer Institute

Prof. Tyson Valentine Sharp
Centre Lead
Centre of Molecular and Cellular Biology
Barts Cancer Institute
Old Anatomy Building
Charterhouse Square
London EC1M 6BQ
bci.qmul.ac.uk
Email: t.sharp@qmul.ac.uk

-Kindly include antibody concentration in Table S1 and kindly callout to the table in the methods section. Added

-Please expand on details for imaging include NA for objectives and excitation/emission information. Added page 24, lines 21-23.

-Please add molecular weight markers for Figure S1A. Added

-Please merge supplementary methods information with the main section on methods. Done
-Please provide sequence information for the targeting of exon 1 and related information for siRNA. Added page 20, lines 16-18.

-Please elaborate on the source of PBMCs in the PBMC-co-culture experiments. Kindly provide the necessary declaration for harvesting these cells. Added page 27, lines 5-7

-Kindly elaborate on the purified proteins used for the in-vitro Ubiqiuitnation Assays or call out to the relevant section if already provided. Added page 9, line 26

-Please include callout for: Table S1 called out on page 20 line 22 and page 21 line 27, Figures 2I-J (figures 2I-J don't exist), 3J already called out page 9 line 19 and S2C called out on Page 8 line 3.

-Please remove the callout for Figure S2H (which does not exist). Done

-Please upload your main manuscript text as an editable doc file. Done

-Please upload all figure files as individual ones, including the supplementary figure files; all figure legends should only appear in the main manuscript file. Please remove figures from main manuscript file. Done

-Please add your main, supplementary figure, and table legends to the main manuscript text after the references section. Done

-please upload your Supporting Table in editable .doc or excel format. Done

Queen Mary University of London

Barts Cancer Institute

Prof. Tyson Valentine Sharp
Centre Lead
Centre of Molecular and Cellular Biology
Barts Cancer Institute
Old Anatomy Building
Charterhouse Square
London EC1M 6BQ
bci.qmul.ac.uk
Email: t.sharp@qmul.ac.uk

-Please add a Category for your manuscript in our system. Done

-please add Keywords for your manuscript in our system. Done

-Please add a Summary Blurb/Alternate Abstract in our system. Done

-Please add the X and Bluesky handles of your host institute/organization, as well as your own, and/or one of the authors, in our system. Done

-please add ORCID ID for secondary corresponding author--you should have received instructions on how to do so. There was a problem. 0000-0002-4508-9606 is Kunal's ORCID if you could add this please?

-Please be sure that the authorship listing and order are correct and match between the system and manuscript file. Done

-Please consult our manuscript preparation guidelines https://www.life-science-alliance.org/manuscript-prep and make sure your manuscript sections are in the correct order. Done

-Please remove ORCID IDs from manuscript cover page. Done

-Please add an Author Contributions section to your main manuscript text. Done

-Please use the [10 author names et al.] format in your references (i.e., limit the author names to the first 10). Done, citations and References formatted to Life Science Alliance format with EndNote.

-The "Data Availability" section should be placed after the Materials & Methods section. Please consult our guidelines at https://www.life-science-alliance.org/manuscript-prep#format Added

Queen Mary University of London

Barts Cancer Institute

Prof. Tyson Valentine Sharp
Centre Lead
Centre of Molecular and Cellular Biology
Barts Cancer Institute
Old Anatomy Building
Charterhouse Square
London EC1M 6BQ
bci.qmul.ac.uk
Email: t.sharp@qmul.ac.uk

-Please be sure that the authorship listing and order is correct

Yours faithfully,

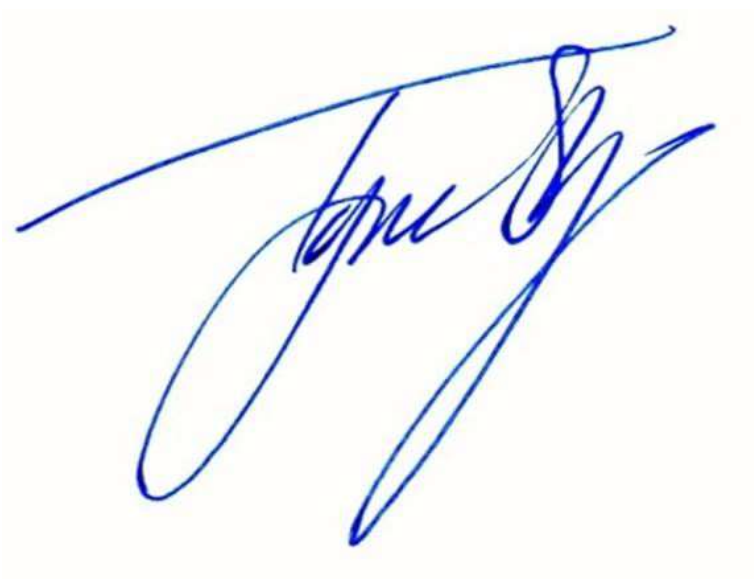

Prof. Tyson V. Sharp

Professor of Cellular and Molecular Biology

July 28, 2026

RE: Life Science Alliance Manuscript #LSA-2026-03812-TR

Prof. Tyson Valentine Sharp
Queen Mary University of London
Centre for Molecular Oncology
Barts Cancer Institute
Queen Mary, University of London
London, Not applicable EC1M 6BQ
United Kingdom

Dear Dr. Sharp,

Thank you for submitting your Research Article entitled "Clonal LIMD1 loss drives PD-L1 immune evasion via ARIH1-dependent ubiquitination in lung cancer" and responding to most of our formatting requests. For one of our points on images of blots in Figures 1A and S1A, we noted that you addressed the same by removing the blot in Figure1A for A549 cells.

Overall, it is a pleasure to let you know that your manuscript is now accepted for publication in Life Science Alliance. Congratulations on this interesting work.

The final published version of your manuscript will be deposited by us to PubMed Central upon publication.

Your manuscript will now progress through copyediting and proofing. At the proofing stage, in alignment with LSA's guidelines, please remove the entire section (line 62) on 'Significance Statement' from the manuscript.

The final published version of your manuscript will be deposited by us to PubMed Central upon publication.

Your manuscript will now progress through copyediting and proofing. It is journal policy that authors provide original data upon request.

Your article will publish open access upon publication under a CC-BY license.

Reviews, decision letters, and point-by-point responses associated with peer-review at Life Science Alliance will be published, alongside the manuscript. If you do want to opt out of having the reviewer reports and your point-by-point responses displayed, please let us know immediately.

IMPORTANT: If you will be unreachable at any time, please provide us with the email address of an alternate author. Failure to respond to routine queries may lead to unavoidable delays in publication.

Scheduling details will be available from our production department. You will receive proofs shortly before the publication date. Only essential corrections can be made at the proof stage so if there are any minor final changes you wish to make to the manuscript, please let the journal office know now.

DISTRIBUTION OF MATERIALS:
Authors are required to distribute freely any materials used in experiments published in Life Science Alliance. Authors are encouraged to deposit materials used in their studies to the appropriate repositories for distribution to researchers.

You can contact the journal office with any questions, contact@life-science-alliance.org

Again, congratulations on a very nice paper. I hope you found the review process to be constructive and are pleased with how the manuscript was handled editorially. We look forward to future exciting submissions from your lab.

Sincerely,

Sarita Hebbar, PhD
Scientific Editor
Life Science Alliance
http://www.lsajournal.org
